# Acute associations between heatwaves and preterm and early-term birth in 50 US metropolitan areas: a matched case-control study

**DOI:** 10.1186/s12940-021-00733-y

**Published:** 2021-04-23

**Authors:** Mengjiao Huang, Matthew J. Strickland, Megan Richards, Heather A. Holmes, Andrew J. Newman, Joshua V. Garn, Yan Liu, Joshua L. Warren, Howard H. Chang, Lyndsey A. Darrow

**Affiliations:** 1 School of Community Health Sciences, University of Nevada, 1664 N. Virginia Street, Reno, NV 89557 USA; 2 Department of Chemical Engineering, University of Utah, Salt Lake City, UT USA; 3 National Center for Atmospheric Research, Boulder, CO USA; 4 Department of Biostatistics, Yale University, New Haven, CT USA; 5 Rollins School of Public Health, Emory University, Atlanta, GA USA

**Keywords:** Early-term birth, Climate change, Heatwave, Preterm birth

## Abstract

**Background:**

The effect of heatwaves on adverse birth outcomes is not well understood and may vary by how heatwaves are defined. The study aims to examine acute associations between various heatwave definitions and preterm and early-term birth.

**Methods:**

Using national vital records from 50 metropolitan statistical areas (MSAs) between 1982 and 1988, singleton preterm (< 37 weeks) and early-term births (37–38 weeks) were matched (1:1) to controls who completed at least 37 weeks or 39 weeks of gestation, respectively. Matching variables were MSA, maternal race, and maternal education. Sixty heatwave definitions including binary indicators for exposure to sustained heat, number of high heat days, and measures of heat intensity (the average degrees over the threshold in the past 7 days) based on the 97.5^th^ percentile of MSA-specific temperature metrics, or the 85^th^ percentile of positive excessive heat factor (EHF) were created. Odds ratios (OR) for heatwave exposures in the week preceding birth (or corresponding gestational week for controls) were estimated using conditional logistic regression adjusting for maternal age, marital status, and seasonality. Effect modification by maternal education, age, race/ethnicity, child sex, and region was assessed.

**Results:**

There were 615,329 preterm and 1,005,576 early-term case-control pairs in the analyses. For most definitions, exposure to heatwaves in the week before delivery was consistently associated with increased odds of early-term birth. Exposure to more high heat days and more degrees above the threshold yielded higher magnitude ORs. For exposure to 3 or more days over the 97.5^th^ percentile of mean temperature in the past week compared to zero days, the OR was 1.027 for early-term birth (95%CI: 1.014, 1.039). Although we generally found null associations when assessing various heatwave definitions and preterm birth, ORs for both preterm and early-term birth were greater in magnitude among Hispanic and non-Hispanic black mothers.

**Conclusion:**

Although associations varied across metrics and heatwave definitions, heatwaves were more consistently associated with early-term birth than with preterm birth. This study’s findings may have implications for prevention programs targeting vulnerable subgroups as climate change progresses.

**Supplementary Information:**

The online version contains supplementary material available at 10.1186/s12940-021-00733-y.

## Background

Preterm birth (birth less than 37 weeks of gestational age) is a leading cause of infant mortality and long-term neurological disabilities in children [1]. Children born preterm are more likely to experience other adverse outcomes, such as respiratory illnesses [2], lower cognitive abilities [3, 4], and increased behavioral problems [3, 4]. Maternal and neonatal adverse outcome rates reach the lowest point between 39 and 40 weeks gestation [5, 6]. Early-term birth (37–38 weeks of gestational age) also poses a measurable impact on an infant’s survival, growth, and development relative to those born at 39–40 weeks [5, 6]. In the United States (US) during 1982–1988, about 9.8% of live births were born preterm and 17.8% were born early-term [7]. In 2018, while there was a slight increase in preterm births (10%), early-term births increased significantly, affecting 26.5% of live births [8]. Despite this public health relevance, early-term birth has been understudied.

Recent studies suggest that higher ambient temperatures are associated with an increase in preterm birth [9–16] and a decrease in mean gestational age [17, 18]. However, the magnitude of association varied considerably, and it is unclear what factors influence this relationship. A few studies also found that heatwaves, conceptually defined as consecutive days of high ambient temperature, were positively associated with preterm birth in the last week before delivery [19–22], and that these associations persisted even after adjusting for current day temperatures [23]. Unfortunately, synthesizing results from heatwave studies is challenging due to differences in location, study design, and heatwave definition.

Broadly, there are two major frameworks for defining heatwaves commonly used in the literature. One widely used framework is based on a temperature metric (e.g., maximum temperature, apparent temperature) exceeding a pre-specified threshold, which can be absolute (e.g., 35 °C) or relative (e.g., 95^th^ percentile) for more than a certain number of consecutive days [20, 24]. The other prominent heatwave definition framework, known as excessive heat factor (EHF) [25], has also been used in several studies [26–28]. EHF is a compound value that identifies and quantifies three-day periods in relation to both the historical 95^th^ percentile of temperature and to temperatures over the past 30 days. The choice of temperature metrics may be important, because different metrics measure different aspects of heatwaves. For example, minimum temperatures may reflect the lack of overnight cooling [29], whereas apparent temperature incorporates humidity, which may better reflect how hot it feels outside [30]. It is unclear which temperature metric may be most relevant for adverse health outcomes. Few studies have systematically evaluated how the choice of temperature metrics and threshold of consecutive days affects estimated associations [20]. In addition, previous US studies of high temperatures and preterm birth have focused on single study cites [15, 31, 32], which have limited ability to investigate more extreme regional heatwave events.

To address these literature gaps, we used US birth data from 1982 to 1988 to assess how various heatwave definitions using temperature metrics, the heatwave definition framework, and the number of consecutive hot days were each associated with preterm and early-term birth in the 50 most populous metropolitan areas.

## Methods

### Study population and locations

**Fig. 1:**
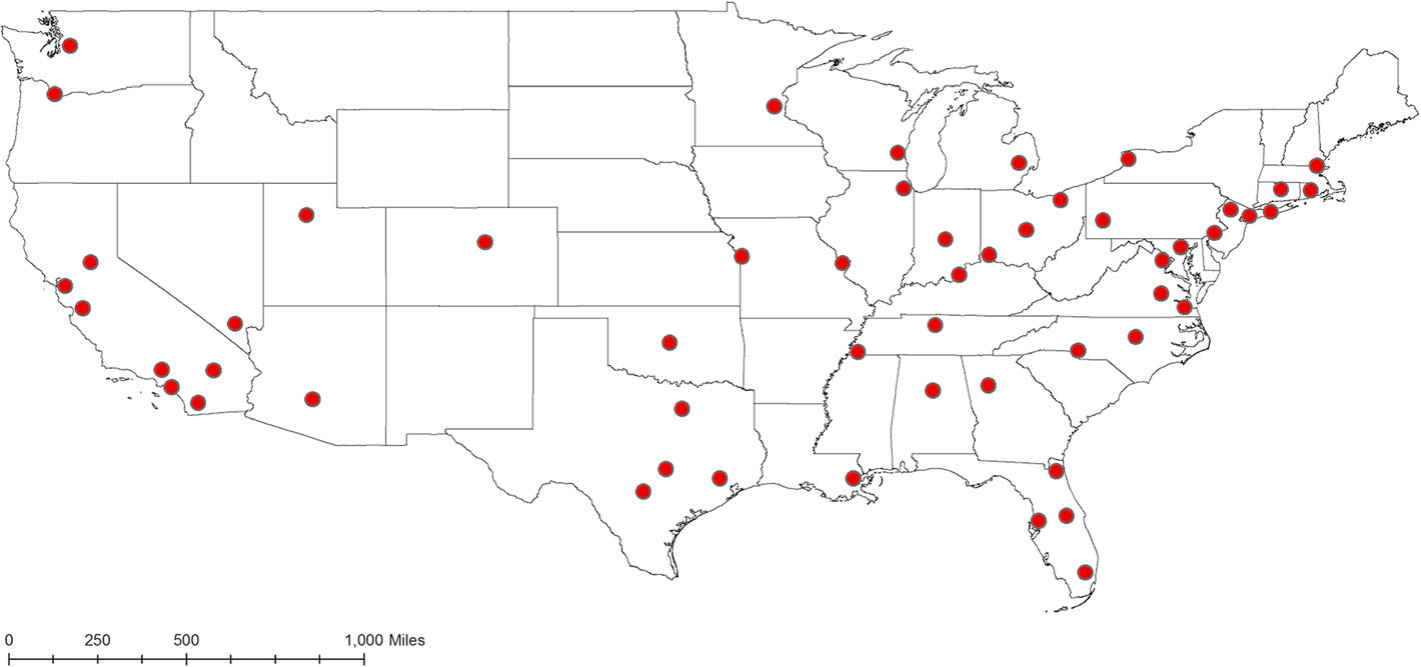
Map of geographic centroids of 53 selected study locations in the US

Data from all births in the US were obtained from the National Center for Health Statistics (NCHS, https://www.cdc.gov/nchs/data_access/vitalstatsonline.htm) for the years 1982–1988, years for which location and exact birth date are publicly available. A total of 25,328,335 births occurred during 1982–1988. We selected births from the 50 most populous metropolitan statistical areas (MSA), accounting for 52% of all births in the US. Delineation of MSAs was based on the 2010 Census. The 50 MSAs contained 416 counties. 20% of births were in the two most populous MSAs (Los Angeles-Long Beach-Santa Ana, CA and New York-Northern New Jersey-Long Island, NY-NJ-PA); because of the disproportionate number of births in those two MSAs, we split them into smaller areas. Los Angeles-Long Beach-Santa Ana, CA was split into two parts (Los Angeles county and Orange county, Figure S1), and New York-Northern New Jersey-Long Island, NY-NJ-PA was split into three parts (Long Island areas, New York City area, and other New York-New Jersey counties, Figure S2). Thus, there were 53 MSA/sub-MSAs (Fig. 1) included in the study; throughout, we refer to these MSAs and sub-MSAs as the “study locations.” The study protocol was approved by the University of Nevada, Reno Institutional Review Board (IRB#: 1164285–8).

### Exclusion criteria

Fixed cohort bias can occur in retrospective birth cohort studies that define inclusion based on birth dates within a fixed start and end date, which means shorter pregnancies are missed at the start of the study, and longer pregnancies are missed at the end [33]. To avoid this bias, we defined our study population by conception dates and limited our study population to women whose last menstrual period (LMP) dates were between October 1, 1981 and February 29, 1988 to ensure that both the shortest and the longest pregnancies were included. For comparison with previous studies, we focussed our analysis on the warm season [9, 16]. Analogous to the fixed cohort bias described above, selection bias might also occur in this setting when shorter or longer pregnancies, which could have been exposed to heatwaves and were at risk for preterm birth, are excluded as a consequence of limiting the analysis to babies born in the warm season. To prevent this potential bias, we included all births whose at-risk window (28 weeks and 0 days − 36 weeks and 6 days for preterm and 37 weeks and 0 days-38 weeks and 6 days for early-term) overlapped with the warm season (May 1 – September 30) for at least 1 day (calculation illustrated in Figure S3). This corresponded to yearly conception dates between August 16 and March 18 (of the following year) and August 2 and January 14 (of the following year) for preterm and early-term birth, respectively (selection process and study flow diagram illustrated in Figure S4). In addition, we limited the analysis to singleton births.

### Meteorological data

We obtained 1 km × 1 km gridded estimates of daily meteorological parameters from Daymet [34] supported by NASA through the Earth Science Data and Information System (ESDIS) and the Terrestrial Ecology Program. Daymet uses surface meteorological observations in a spatial interpolation algorithm that also incorporates elevation, solar radiation, and precipitation factors to provide the gridded estimates. It provides seven surface weather parameters (minimum temperature (Tmin), maximum temperature (Tmax), precipitation, shortwave radiation, vapor pressure (VP), snow water equivalent, and day length) for North America. For this study, we calculated county-level daily Tmin, Tmax, and VP by averaging all grid estimates within a county in the US for each day between January 1, 1982 and December 31, 1988. Daily mean temperature (Tmean) was the mean of daily Tmin and Tmax. Because relative humidity is a qualitative indicator of moisture in the atmosphere that changes with ambient temperature, we instead use dew-point temperature, which is a quantitative measure of the amount of moisture. To provide a humidity value, dew-point temperature was calculated from Tmean and VP [35, 36]. We also calculated apparent temperature (AT) metrics including ATmin, ATmax, ATmean as follows [30]:

1 $$ \mathrm{AT}=-2.653+0.994\times {T}_a+0.0153\times {T}_d^2, $$

where *T*_*a*_ is ambient temperature (minimum, maximum, or mean) and *T*_*d*_ is mean dew-point temperature.

For study locations comprised of more than one county, we calculated daily meteorological variables for each location by linking county-level population size obtained from the 1980 Census (https://www2.census.gov/programs-surveys/popest/tables/1980-1990/counties/totals/e8089co.xls) to the meteorological data and calculating population-weighted mean of the county-level estimates within a location.

### Heatwave definitions

**Fig. 2:**
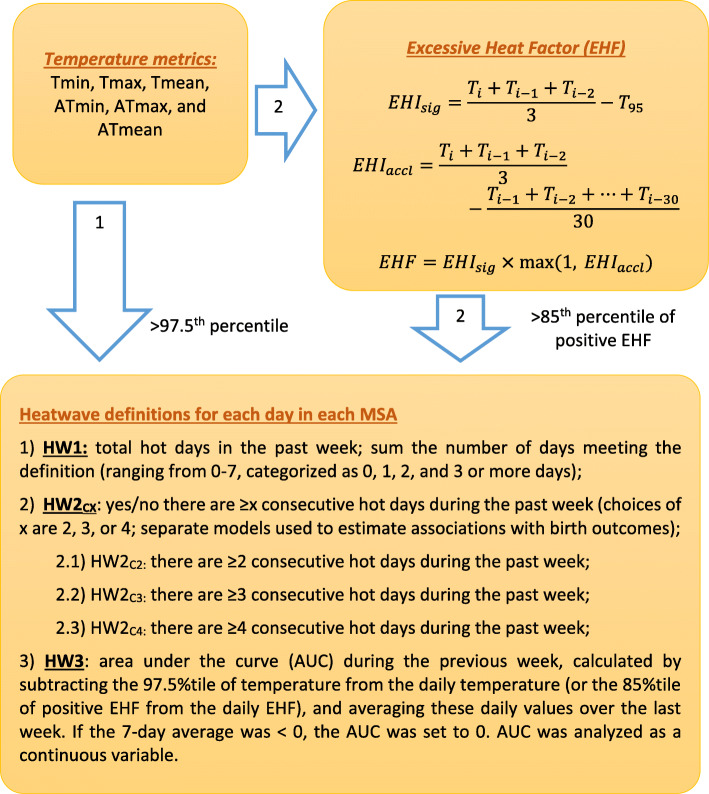
Illustration of heatwave definitions. Sixty heatwave variables were generated with the combination of temperature metrics and heatwave definitions listed in the figure

We used two major heatwave definition frameworks (Fig. 2) from the existing literature to identify hot days in each location over the study period. One framework (Arrow 1, Fig. 2) is referred to as the relative temperature threshold. Because climate differs across study locations, we set temperature thresholds specific to each study location [37]. Since this was a national study with a relatively large sample size, we were able to examine the impact of more extreme heatwave events. Therefore, we used the 97.5^th^ percentile [20, 38–40] (denoted as T_97.5_) as the cut-off value for six temperature metrics: Tmin, ATmin, Tmax, ATmax, Tmean, and ATmean.

The other framework (Arrow 2, Fig. 2) is the excessive heat factor (EHF) [25], which is derived from the temperature metrics using formulas 2–4. In addition to performing these calculations using mean air temperature, we also performed them using Tmin, ATmin, Tmax, ATmax, and ATmean. We used the relative threshold recommended by Nairn and Facett to define extreme heatwave (85^th^ percentile of all positive EHF, denoted as EHF^+^ _85_). Specifically, the calculations are given as

2 $$ EH{I}_{sig}=\frac{T_i+{T}_{i-1}+{T}_{i-2}}{3}-{T}_{95}, $$

where $\frac{T_i+{T}_{i-1}+{T}_{i-2}}{3}$ is the 3-day average of daily temperature and *T*_95_ is the 95^th^ percentile (location-specific) of daily temperature for the climate reference period 1982–1988;

3 $$ EH{I}_{accl}=\frac{T_i+{T}_{i-1}+{T}_{i-2}}{3}-\frac{T_{i-1}+{T}_{i-2}+\cdots +{T}_{i-30}}{30}, $$

where EHI_accl_ is the difference between 3-day average temperature and 30-day average temperature; and

4 $$ EHF= EH{I}_{sig}\times \max \left(1, EH{I}_{accl}\right), $$

where the unit of EHF is °C^2^.

Heatwave indicators were linked to women based on their MSA codes and gestational exposure window. Using the above 12 indicators for hot days (6 temperature metrics × 2 thresholds: T_97.5_ or EHF^+^ _85_), we operationalized heatwave exposures in each location on each day in three different ways (Fig. 2):

1. HW1: focuses on the total number of single hot days in the previous week (sum of the number of days exceeding the threshold (ranging from 0 to 7), categorized as 0, 1, 2, and 3 or more days, 3–7 days were grouped together to gain more precision;
2. HW2_CX_: emphasizes the impact of sustained heat, indicator variables for ≥x consecutive (denoted as subscript CX) hot days in the past week (choices of x are 2, 3, or 4; separate models were used to estimate associations with birth outcomes);
3. HW3: area under the curve (AUC) during the previous week is a continuous metric incorporating both intensity and duration, calculated by subtracting the T_97.5_ threshold value from the daily temperature (or the EHF^+^ _85_ value from the daily EHF) and averaging these daily differences over the last week. If the 7-day average was less than 0, the AUC was set to 0. AUC values were analyzed as a non-negative continuous variable.

### Statistical analysis

We used a 1:1 matched case-control design with cumulative sampling [41], which enabled us to align the exposure window in the case (the 7 days preceding birth) to the corresponding gestational week in the control. We selected controls from births that were > 36 weeks and > 38 weeks by the time of delivery for preterm and early-term birth cases, respectively. Matching factors were maternal race/ethnicity and education, which are strong predictors of preterm birth and early-term birth, as well as location. Since all subjects in the same location would be assigned the same exposure on a given day, the exposure contrasts in the study were temporal. Individuals with missing information on these factors (maternal race/ethnicity and education) were also matched; all individuals included in the analysis had complete data for location and gestational age. Odds ratios (OR) for HW1, HW2_c2_, HW2_c3_, HW2_c4_, and HW3 were estimated using conditional logistic regression adjusting for maternal age (≤20, 21–34, and ≥ 35 years) [14, 21], marital status (married and unmarried), and LMP month and year (Eq.5). To retain every sampled pregnancy in the analysis, we created an indicator variable for missing values of marital status (0.03% of the sample). The exposure period for each case was the 7 days leading up to birth; the same 7-day gestational period was used as the exposure period for each matched control. Detailed model specifications for each heatwave indicator are presented in Table S1. HW1 was modeled as a categorical variable (0 (reference), 1, 2, and 3 or more days (Table S1); to test for a linear trend, these categories were modeled using a linear term (Table S1); HW2_CX_ was modeled as a binary variable (1 = there was a ≥ X (where X = 2, 3, or 4) consecutive-day heatwave in the past week, 0 = otherwise, Table S1); and HW3 was modeled as a continuous variable (°C for temperature or °C^2^ for EHF, Table S1) .

To examine possible effect modification by maternal characteristics on the associations of heatwaves with preterm and early-term births, we conducted analyses stratified by maternal education (< 12 years, 12 years, and ≥ 13 years), maternal race/ethnicity (Hispanic, non-Hispanic white, and non-Hispanic black), child sex (male and female), and region (Northeast, Midwest, South, and West) and tested interaction by adding an interaction term with heatwave indicators to our primary model (Table S1). To examine the robustness of our results, instead of matching on location and adjusting for LMP month and year (as was done in the primary analysis), we instead matched on LMP month and year and adjusted for location. In addition, we conducted a sensitivity analysis restricted to complete observations (i.e., excluding observations missing matching factors or covariates). All statistical analyses were conducted using SAS (SAS version 9.4; SAS Institute Inc., Cary, NC).

## Results

Table 1 shows descriptive statistics for heatwave indicators using the various definitions. The frequency of heatwaves was highly dependent on the choice of heatwave indicator. For example, having 1 day in the past week above the 97.5^th^ percentile (HW1) was 18 times more common than having 4 or more consecutive days above the 85^th^ percentile for EHF (HW2). In general, the 97.5^th^ percentile of temperature metrics (T_97.5_) resulted in more heatwave days compared to the 85^th^ percentile of positive EHF (EHF^+^ _85_). For example, the mean number of days per year (across years and locations) which had 3 or more days over the threshold in the past week was between 8.5–9.9 days (depending on the temperature metric when T_97.5_ was used), whereas it ranged between 2.0–2.4 days for EHF^+^ _85_ (Table 1). For the most extreme heatwave definition (≥4 consecutive days above the threshold), there were 3.2–4.4 days per year per location using T_97.5_ compared to 0.8–1.2 days per year per location using EHF^+^ _85_. The mean area under the curve (AUC) for EHF^+^ _85_ was larger than T_97.5_ due to the inflation factor by extreme temperature relative to the past 30 days (i.e., EHI_accl_) in the EHF calculation. The mean AUC was between 0.52–0.91 °C and 1.2–3.8°C^2^ for T_97.5_ and EHF^+^ _85_, respectively, varying by temperature metrics used (Table 1). Fewer extreme events were found using minimum temperature (Tmin and ATmin) compared to maximum temperature and mean temperature. For instance, on average, there were 14.6 days per year with ≥2 consecutive days over T_97.5_ in the past week using minimum temperature and 16.0 days per year using maximum temperature (Table 1).

**Table 1:** Mean number of days per year defined as heatwave days (across 53 locations) for various heatwave definitions

	97.5^th^ percentile of temperature						85^th^ percentile of EHF (+)					
	Tmin	ATmin	Tmax	ATmax	Tmean	ATmean	Tmin	ATmin	Tmax	ATmax	Tmean	ATmean
**HW1 (n**^**a**^**, SD**^**b**^**)**												
0	334.4 (3.7)	334.4 (3.8)	337.7 (4.3)	338.2 (4.2)	338.0 (4.0)	337.0 (4.0)	357.3 (1.5)	357.5 (1.5)	357.1 (1.8)	357.1 (1.6)	357.1 (1.9)	357.4 (1.8)
1	14.2 (3.4)	14.3 (3.5)	10.7 (3.4)	10.3 (3.2)	10.3 (3.2)	11.6 (3.3)	3.7 (1.4)	3.8 (1.5)	3.1 (1.3)	3.3 (1.5)	3.3 (1.6)	3.3 (1.5)
2	7.8 (2.0)	8.0 (2.0)	7.0 (2.2)	7.2 (2.3)	7.2 (2.2)	7.4 (2.0)	2.3 (0.9)	2.0 (1.0)	2.8 (1.2)	2.6 (1.0)	2.6 (1.2)	2.4 (1.3)
3 or more	8.9 (1.5)	8.5 (1.5)	9.9 (2.0)	9.6 (1.5)	9.9 (1.6)	9.3 (1.5)	2.0 (0.6)	2.0 (0.6)	2.3 (1.0)	2.3 (0.9)	2.4 (0.9)	2.2 (0.9)
**HW2 (n**^**a**^**, SD**^**b**^**)**												
≥ 2 consec. Days	14.6 (1.8)	14.6 (1.8)	16.0 (2.0)	16.1 (2.2)	16.1 (2.4)	15.4 (2.3)	4.2 (0.9)	4.0 (1.1)	5.0 (1.1)	4.8 (0.9)	4.8 (1.1)	4.5 (0.9)
≥ 3 consec. Days	6.9 (1.5)	6.5 (1.6)	8.7 (1.9)	8.2 (1.5)	8.4 (1.5)	7.8 (1.5)	1.9 (0.6)	1.9 (0.6)	2.3 (1.0)	2.3 (0.9)	2.3 (0.8)	2.1 (0.9)
≥ 4 consec. Days	3.2 (1.2)	3.2 (1.1)	4.4 (1.6)	4.2 (1.5)	4.3 (1.5)	3.8 (1.3)	0.8 (0.4)	0.8 (0.3)	1.0 (0.4)	1.2 (0.5)	1.0 (0.5)	0.9 (0.4)
**HW3**												
n^a^(SD^b^)	3.3 (1.2)	3.3 (1.1)	4.5 (1.8)	4.1 (1.5)	4.3 (1.4)	4.0 (1.2)	0.9 (0.4)	0.9 (0.5)	1.2 (0.6)	1.1 (0.6)	1.0 (0.5)	0.9 (0.5)
AUC (°C/°C^2^, mean (SD^c^))	0.52 (0.52)	0.78 (0.81)	0.76 (0.67)	0.91 (0.78)	0.57 (0.51)	0.83 (0.80)	1.2 (1.1)	2.4 (2.3)	3.7 (4.3)	3.8 (4.1)	1.4 (1.5)	2.6 (2.7)

Note: Excessive heat factor (EHF); HW1: the total number of hot days in the previous week (sum the number meeting the definition (ranging from 0 to 7)); HW2: number of consecutive hot days in the past week (binary indicators for 2, 3 and 4 consecutive days); HW3: area under the curve (AUC) during the previous week

^a^ n is the mean annual number of days meeting the exposure definition across 53 locations

^b^ Standard deviation (SD), describes the variation of number of days per year across 53 locations

^c^ Standard deviation (SD), describes the variation of area under the curve (AUC) across 53 locations and all years

1,005,576 case-control pairs were included in the early-term birth analysis. Most early-term matching pairs were non-Hispanic white (59.0%); 28.8% of mothers completed exactly 12 years of education, and 30.8% were from southern study locations (Table 2). Proportions of risk factors that were not matched on, such as mothers age ≥ 35 years and proportion unmarried, were higher among cases (8.7 and 26.3%, respectively) than among controls (7.1 and 25.9%, respectively; Table 2).

**Table 2:** Maternal and child characteristics of 1: 1 matched cases and controls for preterm birth (PTB) and early-term birth (ETB), 1982–1988

Outcome	Preterm birth assessment		Early-term birth assessment	
	PTB Case (*N* = 615,329)	Control (*N* = 615,329)	ETB Case (*N* = 1,005,576)	Control (*N* = 1,005,576)
**Maternal race/ethnicity,** n (%)				
Hispanic	88,103 (14.3)	88,103 (14.3)	137,747 (13.7)	137,747 (13.7)
Non-Hispanic white	299,096 (48.6)	299,096 (48.6)	593,439 (59.0)	593,439 (59.0)
Non-Hispanic black	202,327 (32.9)	202,327 (32.9)	225,067 (22.4)	225,067 (22.4)
Other	23,868 (3.9)	23,868 (3.9)	46,115 (4.6)	46,115 (4.6)
Missing	1935 (0.3)	1935 (0.3)	3208 (0.3)	3208 (0.3)
**Maternal years of education,** n (%)				
≤ 12	130,493 (21.2)	130,493 (21.2)	150,163 (14.9)	150,163 (14.9)
12	178,579 (29.0)	178,579 (29.0)	289,889 (28.8)	289,889 (28.8)
≥ 13	131,850 (21.4)	131,850 (21.4)	280,117 (27.9)	280,117 (27.9)
Missing	174,407 (28.4)	174,407 (28.4)	285,407 (28.4)	285,407 (28.4)
**Region of residence,** n (%)				
Northeast	141,127 (23.0)	141,127 (23.0)	245,385 (24.4)	245,385 (24.4)
Midwest	135,587 (22.0)	135,587 (22.0)	222,753 (22.2)	222,753 (22.2)
South	207,442 (33.7)	207,442 (33.7)	310,046 (30.8)	310,046 (30.8)
West	131,173 (21.3)	131,173 (21.3)	227,392 (22.6)	227,392 (22.6)
**Maternal age (year),** n (%)				
≤ 20	109,251 (17.8)	94,273 (15.3)	117,589 (11.7)	120,210 (12.0)
21–34	458,365 (74.5)	479,083 (77.9)	800,633 (79.6)	813,498 (80.9)
≥ 35	47,713 (7.7)	41,973 (6.8)	87,354 (8.7)	71,868 (7.1)
**Child sex,** n (%)				
Male	328,851 (53.4)	313,212 (50.9)	533,361 (53.0)	507,237 (50.4)
**Marital status,** n (%)				
Married	375,376 (61.0)	411,275 (66.8)	740,573 (73.7)	745,228 (74.1)
Unmarried	239,779 (39.0)	203,842 (33.1)	264,718 (26.3)	260,062 (25.9)
Missing	174 (0.03)	212 (0.03)	285 (0.03)	286 (0.03)
**Month of last normal menses,** n (%)				
January	80,971 (13.2)	88,654 (14.4)	84,520 (8.4)	91,597 (9.1)
February	88,635 (14.4)	82,874 (13.5)	0^a^	0^a^
March	40,652 (6.6)	44,981 (7.3)	0^a^	0^a^
August	39,018 (6.3)	36,480 (5.9)	153,484 (12.3)	153,115 (15.2)
September	78,975 (12.8)	76,831 (12.5)	163,803 (16.3)	162,379 (16.2)
October	97,016 (15.8)	95,043 (15.4)	198,497 (19.7)	199,641 (19.9)
November	96,203 (15.6)	92,861 (15.1)	198,761 (19.8)	193,311 (19.2)
December	93,859 (15.3)	97,677 (15.9)	206,511 (20.5)	205,533 (20.4)

Note: Matching factors are maternal race/ethnicity, maternal education, and MSA of residence of the mother

^a^ Women whose LMP date between April–July (and February–July for early-term) were not eligible for the study because the at-risk window for preterm birth was not in the warm season

There were 615,329 case-control pairs in the preterm birth analysis. Most case-control pairs in the preterm birth analysis were non-Hispanic white, accounting for 48.6% of the sample, and 29.0% completed exactly 12 years of education (Table 2). A large percentage (33.7%) of preterm cases came from southern study locations (Table 2). The percentage of mothers aged 20–34 years was lower among cases (74.5%) than controls (77.9%), and the proportion of unmarried mothers was higher among cases (39.0%) than controls (33.1%; Table 2).

**Fig. 3:**
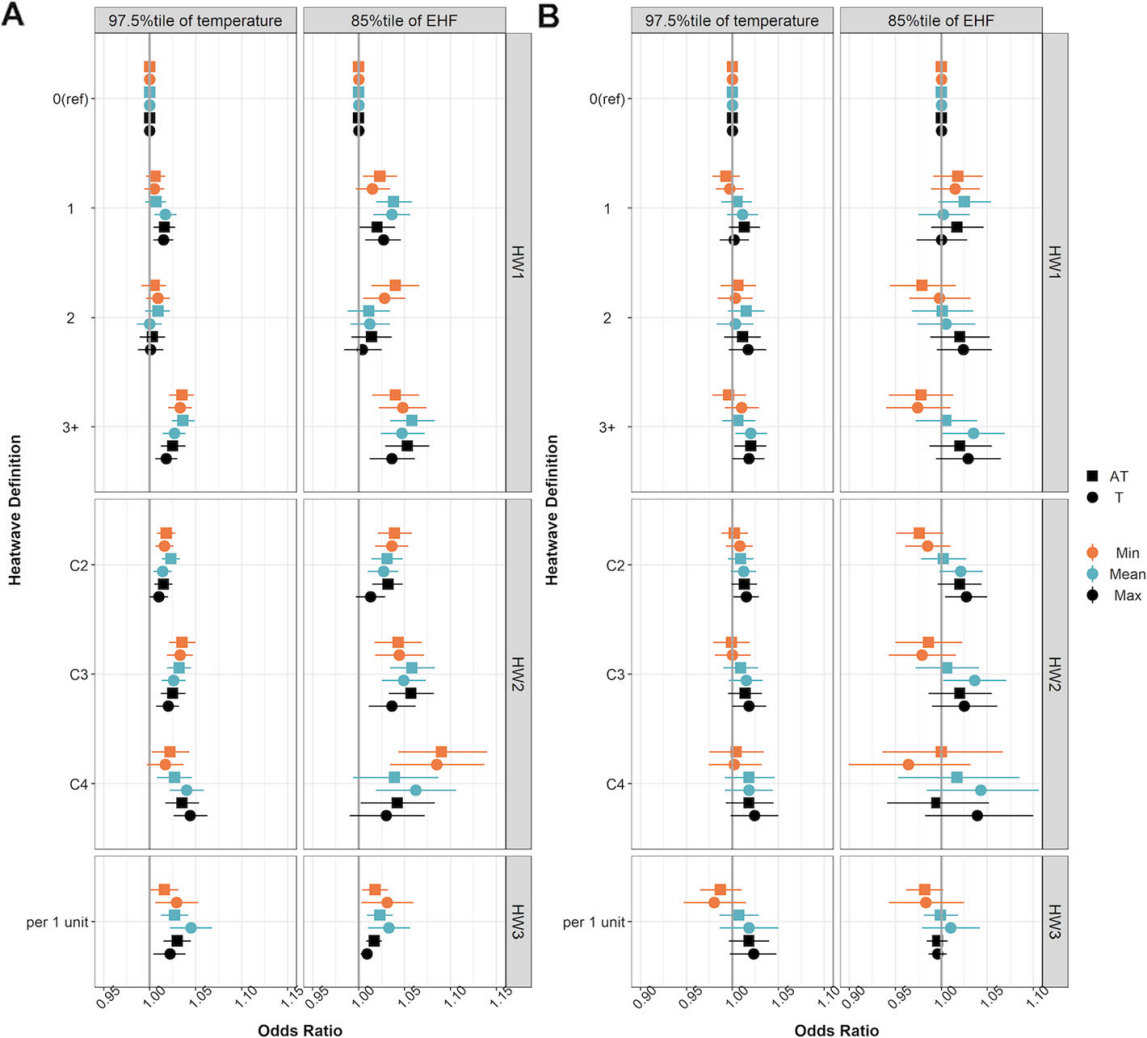
Odds ratio estimates for heatwave indicators: HW1 (top horizontal strip), HW2 (middle strip), and HW3 (bottom strip) for 97.5^th^ percentile for temperature metrics (left column) and 85^th^ percentile of positive EHF for EHF metric (right columns) for early-term (panel A) and preterm birth (panel B). Temperature metrics were grouped based on apparent temperature (AT, square) and temperature (T, solid circle) and minimum (orange), mean (blue), and maximum (black) temperature

In our primary analyses (model refer to Table S1) for sixty heatwave indicators and early-term birth, we found that increases in the duration and intensity (the average degrees over the threshold in the past 7 days) of heatwaves were associated with increased odds of early-term birth. For example, for HW1 (defined as any days exceeding the 97.5^th^ percentile of local Tmean in the 7 days prior to birth), the OR for early-term birth was 1.027 (95%CI, 1.014, 1.039) for mothers who experienced 3 or more hot days during the week before delivery, compared to those who experienced zero hot days (Table S2). We also tested the linear trend across ordinal categories of HW1 (0, 1, 2, and 3 or more days). We observed that the OR increased with an increase in the number of hot days in the previous week (linear trend test, *p* < 0.0001; Table S2). For HW2 (defined as ≥2, ≥3, or ≥ 4 consecutive days of heat in the last 7 days before delivery, evaluated in separate models), we observed a pattern of increasing ORs with increasing heatwave duration: OR = 1.014, 95%CI = 1.004, 1.024 for ≥2 consecutive days; OR = 1.026, 95%CI = 1.013, 1.039 for ≥3 consecutive days; OR = 1.040, 95%CI = 1.022, 1.059 for ≥4 consecutive days (Fig. 3a, Table S2). In addition, for a 1 °C increase in HW3 (average number of degrees that 7-day average Tmean higher than Tmean_97.5_), the OR was 1.045 (95%CI, 1.022, 1.068; Fig. 3a, Table S2). Similar patterns were found when we used other temperature metrics such as Tmin, ATmin, and Tmax, and EHF^+^ _85_ as the threshold for the EHF metric. However, there was larger uncertainty in the associations when using the EHF metric (Fig. 3a, right panel, Table S3) because, in this dataset, EHF^+^ _85_ resulted in a stricter threshold compared to T_97.5_, and consequently, fewer pregnancies were classified as being exposed to a heatwave.

When assessing the association between heatwaves and preterm birth in the primary models (model refer to Table S1), there was less evidence of an association with these heatwave definitions. There was a slight increase in the OR with an increase in HW1 categories (0, 1, 2, and 3 or more days, defined as any days exceeding the 97.5^th^ percentile of local temperature in the 7 days prior to birth) in the week before delivery. For example, when exposed to 1, 2, or 3 or more days with Tmax over Tmax_97.5_ in the week before delivery compared to 0 days, ORs were 1.002 (95%CI, 0.986, 1.018), 1.017 (95%CI, 0.996, 1.037), and 1.018 (95%CI, 1.000, 1.035), respectively (Fig. 3b, Table S4,). However, most effect estimates were consistent with no association.

We compared OR estimates from our primary models using different temperature metrics and EHF to quantify the duration and intensity (the average degrees over the threshold in the past 7 days) of heatwaves for each outcome and found that using apparent temperature produced results similar to those using temperature only (Fig. 3, squares vs. solid circles). In addition, we did not find substantial differences in OR estimates among Tmin (Fig. 3, orange solid circles), Tmean (Fig. 3, blue solid circles), and Tmax (Fig. 3, black solid circles) (or for ATmin, ATmax, and ATmean, also shown in Fig. 3). To examine the robustness of our matched case-control study design, we conducted a sensitivity analysis where instead of matching on study location we matched on LMP month and year and adjusted for study location using indicator variables; the results were similar across heatwave definitions and outcomes (Table S6-S9). We also found similar results to those from our primary analyses after we excluded matched pairs with missing maternal race/ethnicity (0.3%), education (28.4%), and/or marital status (0.03%; Table S10-S11).

**Fig. 4:**
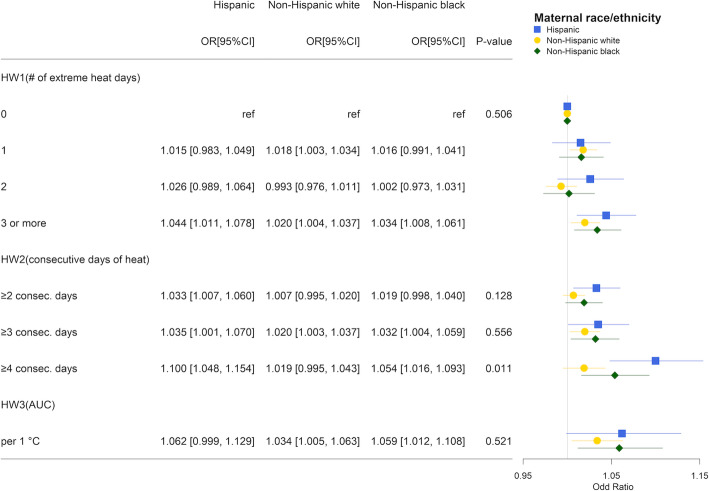
Odds ratio estimates for heatwave indicators on early-term birth by maternal race/ethnicity: Hispanic (blue square), non-Hispanic white (solid yellow circle), and non-Hispanic black (green diamond) using mean temperature over 97.5^th^ percentile to quantify heatwave. *P*-values were obtained from the joint test for interaction

**Fig. 5:**
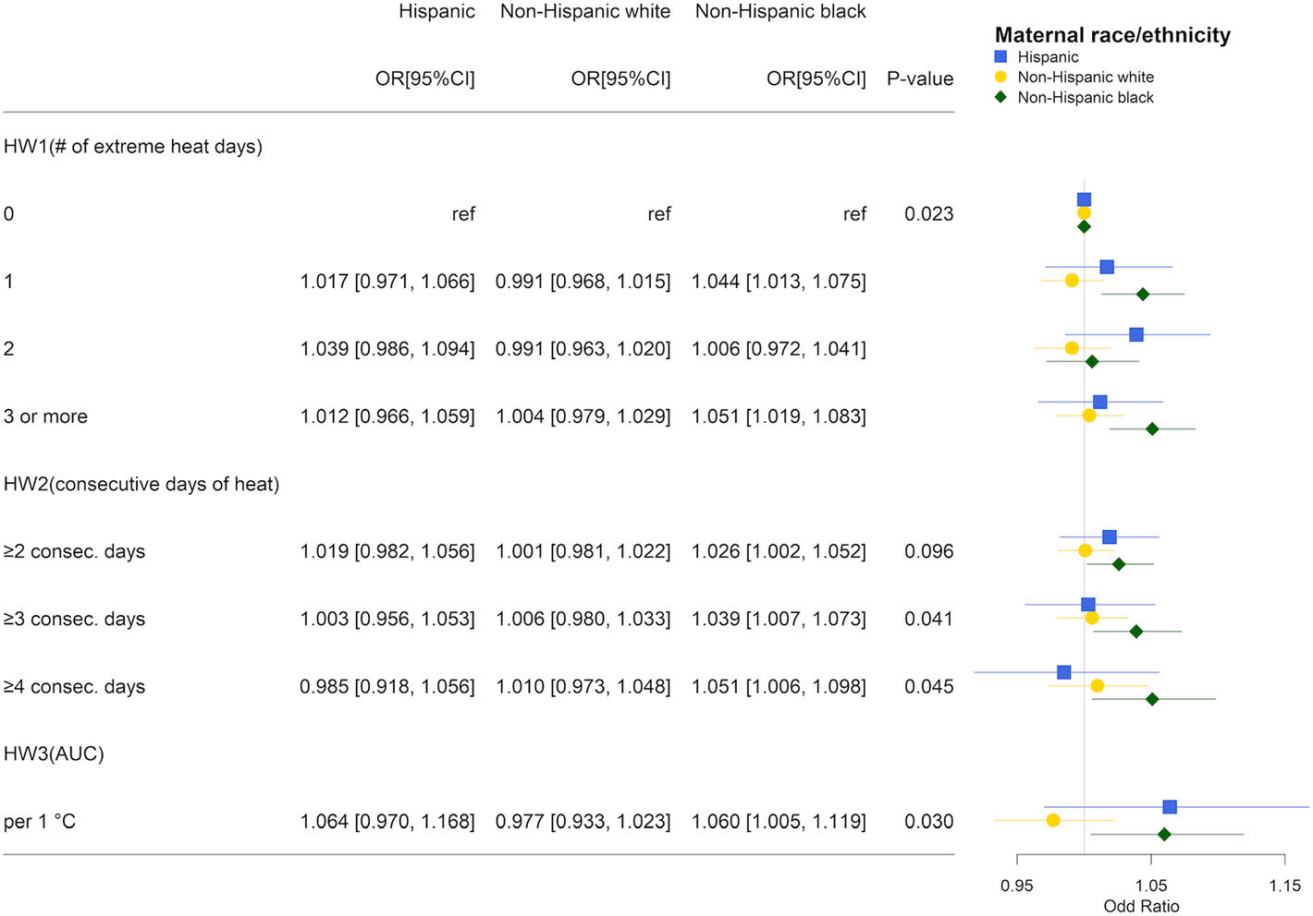
Odds ratio estimates of heatwave indicators on preterm birth by maternal race/ethnicity: Hispanic (blue square), non-Hispanic white (solid yellow circle), and non-Hispanic black (green diamond) using mean temperature over 97.5^th^ percentile to quantify heatwave. *P*-values were obtained from the joint test for interaction

We conducted stratified analyses using the same model specification as our primary models for early-term and preterm matching pairs by race/ethnicity, maternal education, child sex, and region. Large uncertainty was present for stratified analyses using EHF, which had fewer days defined as heatwaves. Thus, we present effect modification results only for Tmean over Tmean_97.5_. Results from the stratified analysis by maternal race/ethnicity suggested that associations of heatwaves and early-term birth tended to be of larger magnitude for Hispanic and non-Hispanic black mothers compared to non-Hispanic white mothers. When exposed to HW2_C4_ (≥4 consecutive days of heat in the week before delivery vs otherwise), the ORs for Hispanic and non-Hispanic black mothers were 1.100 (95%CI 1.048, 1.154) and 1.054 (95%CI, 1.016, 1.093), respectively, whereas the OR for non-Hispanic white mothers was 1.019 (95%CI, 0.995, 1.043; Fig. 4). The joint test for interaction was statistically significant (*p* = 0.011). Although we did not observe a significant overall association with preterm birth, we found a higher OR among Hispanic and non-Hispanic black mothers. For example, per 1 °C increase in HW3 (average number of degrees that 7-day average Tmean higher than Tmean_97.5_), the ORs for Hispanic and non-Hispanic black mothers were 1.064 (95%CI, 0.970, 1.168) and 1.060 (95%CI, 1.005 1.119), respectively, whereas it was 0.977 (95%CI, 0.933, 1.023) for non-Hispanic white mothers (*p* = 0.030 for the joint test for interaction; Fig. 5). In the analyses stratified by maternal education and child sex, no interaction was observed for either preterm birth or early-term birth (Fig. S5-S8). Results from the stratified analysis by region suggested that associations of heatwaves tended to be of larger magnitude in the Northeast and Midwest for early-term birth (Fig. S9), and in the Midwest and South for preterm births (Fig. S10).

## Discussion

In the present study, we developed sixty heatwave indicators to systematically evaluate how parameters in heatwave definitions (i.e., temperature metric and consecutive days) impacted the association between heatwave and preterm and early-term birth using data from 50 metropolitan areas in the US during 1982–1988. Across definitions, exposure to heatwaves in the week before delivery was consistently associated with increased odds of early-term birth. Specifically, we found that exposure to 3 or more single hot days in the past week, ≥3 consecutive days of a sustained heatwave, and increased heatwave intensity (the average degrees over the threshold in the past 7 days) were associated with early-term birth. Associations with preterm birth were weaker compared to early-term birth. Heatwaves defined using maximum temperature showed the most elevated ORs, but the elevation was modest, and most estimates were close to the null. However, effect modification by maternal race/ethnicity was found for both preterm and early-term birth. Specifically, heatwave associations were larger in magnitude among Hispanic and non-Hispanic black mothers compared to non-Hispanic white mothers. In several previous studies, investigators reported positive associations between heatwaves and preterm birth, with larger magnitudes compared to our study [9, 11, 13, 19–22, 42]. In the current study, we observed only a slight overall increase in the risk of preterm birth for certain heatwave definitions, and most estimates were close to the null. Differences might be explained by variation in study design, geographic locations, and time period. One possible explanation might be that our analysis generated estimates of the average effect of heatwaves across multiple states in the US, even though the temperature distributions and population characteristics differed meaningfully across study locations. The overall association might be attenuated by populations less vulnerable to heatwaves.

In the existing literature, early-term birth has received less attention in temperature and heatwave studies. Our findings on early-term birth were consistent with previous findings that exposure to heatwaves or high ambient temperature in the last week before delivery was associated with an increase in the rate of early-term birth [13, 43]. Even so, the magnitude of these associations varied across studies. For example, Auger et al. [43] found a hazard ratio (HR) of 1.27 for early-term pregnancies exposed to 4 to 7 days of 32 °C or higher, while Ha et al. [13] found an OR of 1.04 for temperate exposure above the 90^th^ percentile. Based on our results we expect modest differences in association based on heatwave definition between these studies, but a HR of 1.27 is a considerably higher than any estimate observed in our study.

Heat-related mechanisms are plausible to explain the early onset of labor during extreme heat. Some studies suggest that heat stress increases uterine contraction [44, 45], and dehydration due to heatwaves could reduce uterine blood flow, potentially increasing pituitary hormone levels which induce labor [46]. The sensitivity to heat is greater in late gestation age when thermoregulation may be less efficient [9, 46]. However, no consistent epidemiological evidence suggests that extreme temperature should have a greater impact on births later in gestational age (e.g., late preterm (34–36 weeks) and early-term birth (37–38 weeks)) compared to those in early gestation (e.g., extremely preterm (< 28 weeks) and very preterm (28–32 weeks)). Some studies observed stronger associations of temperature among early preterm birth [11, 14, 19], while others found the opposite [22, 43].

We observed that heatwaves were more strongly associated with both early-term and preterm birth among non-Hispanic black and Hispanic mothers. These findings are in-line with those from previous studies [31, 47, 48]. Minority groups might be more vulnerable to heatwave events due to a lack of a cooling system in the household and less access to neighborhood greenness. According to the American Housing Survey in 1985, about 40% of the homes did not have air conditioners, and this percentage was disproportionately higher among black and Hispanic households [49]. Green spaces in metropolitan cities can help mitigate urban heat islands by absorbing solar radiation and cooling through evapotranspiration and can possibly create differential heat patterns within a city, especially at night [50, 51]. A national-level study found that African-Americans, Asians, and Hispanics were more likely than non-Hispanic whites to live in areas with less tree cover [52]. We did not find large differences in heatwave associations across education levels; however, heatwave associations with early-term birth were somewhat stronger among mothers who had completed less than 12 years of education, consistent with previous studies [9, 11, 16, 53].

Although previous physiological research has shown that high humidity increases the risk of heat-related illness associated with high temperatures [54, 55], when we used apparent temperature (which incorporates humidity) in our analysis, the heatwave associations were similar in magnitude to those using temperature only. This finding is supported by previous epidemiologic studies that found little evidence for improved prediction of mortality and preterm birth by incorporating humidity in the heatwave definitions [20, 56]. Although previous epidemiologic studies have reported a positive association with preterm birth using apparent temperature [9, 32, 57], there is a lack of strong evidence supporting the premise that apparent temperature better predicts preterm birth compared to temperature.

In our study, no single temperature metric best predicted health outcomes. Overall, the use of mean, maximum, minimum temperature, as well as apparent temperature variants, showed similar patterns of associations with the outcomes. When they differed from each other, the associations were not always stronger for a specific temperature metric, indicating that the specific temperature metric might not be an important factor impacting the magnitude of the association between heatwaves and early-term and preterm birth.

Heatwaves defined using EHF were more intense and rare. We observed larger associations in heatwave indicators using EHF with early-term birth; however, due to the rarity of heatwaves based on this framework, estimates were discernibly more uncertain compared to heatwaves defined based on the 97.5^th^ percentile of temperature, even in our large dataset. Broadly, using EHF as a metric to define heatwaves generally produced similar patterns but a larger magnitude in the ORs for most heatwave definitions. Reasons why including an acclimation factor in the calculation might help to identify heatwaves that most strongly impact birth outcomes remains an outstanding question.

This was a nationwide study evaluating the impact of heatwaves on adverse birth outcomes across 50 metropolitan areas covering different climate zones in the US. We systematically evaluated various heatwave definitions to examine how changing those parameters would affect the effect estimates of heatwaves on preterm and early-term birth. To estimate the acute impact of heatwaves while also accounting for seasonality in conception rates, we used a matched case-control study to compare exposure during the same gestational window for cases and controls. To be a confounder in our analysis, a risk factor for these outcomes would have to be correlated with heatwaves, and we controlled tightly for the seasonality and longer-term time trends using indicators for the month of study (based on LMP) to reduce this possibility. The results were robust to matching on LMP month and year instead of location and exclusion of observations with missing covariates.

A study limitation is that our data are from the 1980s, which is some time in the past. Even so, our findings could enable researchers to compare the impact of heatwaves on health changes across decades, when factors such as air conditioning prevalence as well as the frequency, duration, and intensity of heatwaves were different. In addition, we investigated a variety of heatwave definitions, which increases the probability of type one error due to multiple comparisons. However, our interpretations focus on the holistic trends across definitions instead of isolated statistically significant associations. In addition, maternal education was missing for 28% of the birth records. However, maternal education not expected to be a confounder because it is not associated with heatwaves, which lessens concerns about missingness. We also examined the associations of heatwave indicators and preterm and early-term birth among those with missing education; we did not find differences in odds ratios between those missing an education value and other education groups.

## Conclusions

Our results suggest that heatwave duration and intensity are positively associated with an increase in early-term births. The association with preterm birth was slightly elevated for some heatwave definitions, but most of the results suggested no association. However, subgroup analyses by race/ethnicity yielded evidence of positive associations; heatwaves had stronger associations with both early-term and preterm birth for Hispanic and non-Hispanic black mothers, which could help guide intervention programs to mitigate heatwave impacts for vulnerable populations. Our findings suggest that estimated associations based on apparent temperature were similar to those based on temperature. We found no consistent indication that minimum, maximum, or mean temperature was most strongly associated with the outcomes, suggesting that any of these metrics may be appropriate for future research. Finally, relative to the 97.5^th^ percentile threshold cut-off, the EHF measures yielded higher ORs with increased uncertainty.

## Supplementary Information

**Additional file 1: Figure S1.** Map of Los Angeles-Long Beach-Santa Ana MSA. The MSA was split into Los Angeles county (mint green) and Orange county (light orange). **Figure S2.** Map of New York-North New Jersey-Long Island MSA. The MSA was split into the Long Island area (blue), New York City area (pink), and other New York and New Jersey counties (green). **Figure S3.** Illustration of the selection of pregnancies whose at-risk windows overlap with the warm season for preterm (blue) and early-term birth (orange) where pregnancy 1-4 (four arrow lines) are extreme examples determining the range of the eligible LMP dates. The at-risk window was defined as 28 weeks and 0 days to 36 weeks and 6 days for preterm birth and 37 weeks and 0 days to 38 weeks and 6 days for early-term birth. **Figure S4.** Flow chart of identification of preterm and early-term cases and matching control. **Table S1.** Detailed model specification for the analyses of preterm and early-term birth for different types of heatwave indicators. **Table S2.** Odds ratio estimates of heatwave indicators based on definition framework 1 (97.5^th^ percentile of temperature) on early-term birth matching on maternal race, maternal education, and location. **Table S3.** Odds ratio estimates of heatwave indicators based on definition framework 2 (85^th^ percentile of positive excessive heat factor) on early-term birth matching on maternal race, maternal education, and location. **Table S4.** Odds ratio estimates of heatwave indicators based on definition framework 1 (97.5^th^ percentile of temperature) on preterm birth matching on maternal race, maternal education, and location. **Table S5.** Odds ratio estimates of heatwave indicators based on definition framework 2 (85^th^ percentile of positive excessive heat factor) on preterm birth matching on maternal race, maternal education, and location. **Table S6.** Odds ratio estimates of heatwave indicators based on definition framework 1 (97.5^th^ percentile of temperature) on preterm birth matching on maternal race, maternal education, and month and year of the last menstrual period. **Table S7.** Odds ratio estimates of heatwave indicators based on definition framework 2 (85^th^ percentile of positive excessive heat factor) on preterm birth matching on maternal race, maternal education, and month and year of the last menstrual period. **Table S8.** Odds ratio estimates of heatwave indicators based on definition framework 1 (97.5^th^ percentile of temperature) on early-term birth matching on maternal race, maternal education, and month and year of the last menstrual period. **Table S9.** Odds ratio estimates of heatwave indicators based on definition framework 2 (85^th^ percentile of positive excessive heat factor) on early-term birth matching on maternal race, maternal education, and month and year of the last menstrual period. **Table S10.** Odds ratio estimates of heatwave indicators based on definition framework 1 (97.5^th^ percentile of temperature) on preterm birth matching on maternal race, maternal education, and location, excluding those with missing matching factors and covariates. **Table S11.** Odds ratio estimates of heatwave indicators based on definition framework 1 (97.5^th^ percentile of temperature) on early-term birth matching on maternal race, maternal education, and location, excluding those with missing matching factors and covariates. **Figure S5.** Odds ratio estimates of heatwave indicators on early-term birth by maternal education (<12 years, 12 years, and ≥13 years) using mean temperature over 97.5^th^ percentile to quantify heatwave. *P*-values were calculated from the joint test for interaction. **Figure S6.** Odds ratio estimates of heatwave indicators on preterm birth by maternal education (<12 years, 12 years, and ≥13 years) using mean temperature over 97.5^th^ percentile to quantify heatwave. *P*-values were calculated from the joint test for interaction. **Figure S7.** Odds ratio estimates of heatwave indicators on early-term birth by child sex (male and female) using mean temperature over 97.5^th^ percentile to quantify heatwave. *P*-values were calculated from the joint test for interaction. **Figure S8.** Odds ratio estimates of heatwave indicators on preterm birth by child sex (male and female) using mean temperature over 97.5^th^ percentile to quantify heatwave. *P*-values were calculated from the joint test for interaction. **Figure S9.** Odds ratio estimates of heatwave indicators on early-term birth by region (Northeast, Midwest, South, and West) using mean temperature over 97.5^th^ percentile to quantify heatwave. *P*-values were calculated from the joint test for interaction. **Figure S10.** Odds ratio estimates of heatwave indicators on preterm birth by region (Northeast, Midwest, South, and West) using mean temperature over 97.5^th^ percentile to quantify heatwave. *P*-values were calculated from the joint test for interaction.

## Data Availability

The datasets supporting the conclusions of this article are available in the National Center for Health Statistics (NCHS, https://www.cdc.gov/nchs/data_access/vitalstatsonline.htm) and DAYMET (https://daymet.ornl.gov/overview).

## Abbreviations

PTB: preterm birth
ETB: early-term birth
MSA: metropolitan statistical area
US: United States
EHF: excessive heat factor
OR: odds ratio
95%CI: 95% confidence interval
LMP: last menstrual period
NASA: National Aeronautics and Space Administration
ESIDS: Earth Science Data and Information System
Tmin: minimum temperature
Tmax: maximum temperature
Tmean: mean temperature
VP: vapor pressure
ATmin: minimum apparent temperature
ATmax: maximum apparent temperature
ATmean: mean apparent temperature
AUC: area under the curve
HW: heatwave

## References

[1] Goldenberg RL, Culhane JF, Iams JD, Romero R (2008). Epidemiology and causes of preterm birth. Lancet.

[2] Beck S, Wojdyla D, Say L, Betran AP, Merialdi M, Requejo JH (2010). The worldwide incidence of preterm birth: a systematic review of maternal mortality and morbidity. Bull World Health Organ.

[3] Colvin M, McGuire W, Fowlie PW (2004). Neurodevelopmental outcomes after preterm birth. BMJ..

[4] O’Keeffe MJ, O’Callaghan M, Williams GM, Najman JM, Bor W (2003). Learning, cognitive, and attentional problems in adolescents born small for gestational age. Pediatrics..

[5] Spong CY (2013). Defining “term” pregnancy: recommendations from the defining “term” pregnancy workgroup. JAMA..

[6] Stewart DL, Barfield WD. Updates on an at-risk population: late-preterm and early-term infants. Pediatrics. 2019;144(5):e20192760. 10.1542/peds.2019-2760 31636141

[7] National Center for Health Statistics. Detail Natality, 1982-1988. Public-use data file and documentation. National Center for Health Statistics. Available from: https://www.cdc.gov/nchs/data_access/vitalstatsonline.htm. [cited 2018 Sep 27].

[8] Martin JA, Hamilton BE, Osterman MJ, Driscoll AK (2019). Births: final data for 2018.

[9] Basu R, Malig B, Ostro B (2010). High ambient temperature and the risk of preterm delivery. Am J Epidemiol.

[10] Carolan-Olah M, Frankowska D (2014). High environmental temperature and preterm birth: a review of the evidence. Midwifery..

[11] Cox B, Vicedo-Cabrera AM, Gasparrini A, Roels HA, Martens E, Vangronsveld J, Forsberg B, Nawrot TS (2016). Ambient temperature as a trigger of preterm delivery in a temperate climate. J Epidemiol Community Health.

[12] Giorgis-Allemand L, Pedersen M, Bernard C, Aguilera I, Beelen RMJ, Chatzi L, Cirach M, Danileviciute A, Dedele A, van Eijsden M, Estarlich M, Fernández-Somoano A, Fernández MF, Forastiere F, Gehring U, Grazuleviciene R, Gruzieva O, Heude B, Hoek G, de Hoogh K, van den Hooven E, Håberg SE, Iñiguez C, Jaddoe VW, Korek M, Lertxundi A, Lepeule J, Nafstad P, Nystad W, Patelarou E, Porta D, Postma D, Raaschou-Nielsen O, Rudnai P, Siroux V, Sunyer J, Stephanou E, Sørensen M, Eriksen KT, Tuffnell D, Varró MJ, Vrijkotte TG, Wijga A, Wright J, Nieuwenhuijsen MJ, Pershagen G, Brunekreef B, Kogevinas M, Slama R (2017). The influence of meteorological factors and atmospheric pollutants on the risk of preterm birth. Am J Epidemiol.

[13] Ha S, Liu D, Zhu Y, Kim SS, Sherman S, Mendola P (2017). Ambient temperature and early delivery of singleton pregnancies. Environ Health Perspect.

[14] He J-R, Liu Y, Xia X-Y, Ma W-J, Lin H-L, Kan H-D, Lu JH, Feng Q, Mo WJ, Wang P, Xia HM, Qiu X, Muglia LJ (2016). Ambient temperature and the risk of preterm birth in Guangzhou, China (2001–2011). Environ Health Perspect.

[15] Kloog I, Melly SJ, Coull BA, Nordio F, Schwartz JD (2015). Using satellite-based spatiotemporal resolved air temperature exposure to study the association between ambient air temperature and birth outcomes in Massachusetts. Environ Health Perspect.

[16] Schifano P, Lallo A, Asta F, De Sario M, Davoli M, Michelozzi P (2013). Effect of ambient temperature and air pollutants on the risk of preterm birth, Rome 2001–2010. Environ Int.

[17] Dadvand P, Basagana X, Sartini C, Figueras F, Vrijheid M, De Nazelle A (2011). Climate extremes and the length of gestation. Environ Health Perspect.

[18] Ward A, Clark J, McLeod J, Woodul R, Moser H, Konrad C (2019). The impact of heat exposure on reduced gestational age in pregnant women in North Carolina, 2011–2015. Int J Biometeorol.

[19] Ilango S, Weaver M, Benmarhnia T (2019). Extreme heat episodes, wildfires, and risk of preterm delivery in California, 2005-2013. Environ Epidemiol.

[20] Kent ST, McClure LA, Zaitchik BF, Smith TT, Gohlke JM (2013). Heat waves and health outcomes in Alabama (USA): the importance of heat wave definition. Environ Health Perspect.

[21] Wang J, Williams G, Guo Y, Pan X, Tong S (2013). Maternal exposure to heatwave and preterm birth in Brisbane, Australia. BJOG.

[22] Wang Q, Li B, Benmarhnia T, Hajat S, Ren M, Liu T (2020). Independent and combined effects of heatwaves and PM2.5 on preterm birth in Guangzhou, China: a survival analysis. Environ Health Perspect.

[23] Chen K, Wolf K, Hampel R, Stafoggia M, Breitner S, Cyrys J, Samoli E, Andersen ZJ, Bero-Bedada G, Bellander T, Hennig F, Jacquemin B, Pekkanen J, Peters A, Schneider A (2018). Does temperature-confounding control influence the modifying effect of air temperature in ozone–mortality associations?. Environ Epidemiol..

[24] Anderson GB, Bell ML (2010). Heat waves in the United States: mortality risk during heat waves and effect modification by heat wave characteristics in 43 US communities. Environ Health Perspect.

[25] Nairn JR, Fawcett RG (2013). Defining heatwaves: heatwave defined as a heat-impact event servicing all community and business sectors in Australia.

[26] Borg M, Nitschke M, Williams S, McDonald S, Nairn J, Bi P (2019). Using the excess heat factor to indicate heatwave-related urinary disease: a case study in Adelaide. South Australia Int J Biometeorol.

[27] Scalley BD, Spicer T, Jian L, Xiao J, Nairn J, Robertson A, Weeramanthri T (2015). Responding to heatwave intensity: excess heat factor is a superior predictor of health service utilisation and a trigger for heatwave plans. Aust N Z J Public Health.

[28] Varghese BM, Hansen A, Nitschke M, Nairn J, Hanson-Easey S, Bi P, Pisaniello D (2019). Heatwave and work-related injuries and illnesses in Adelaide, Australia: a case-crossover analysis using the excess heat factor (EHF) as a universal heatwave index. Int Arch Occup Environ Health.

[29] Oke TR (1982). The energetic basis of the urban heat island. Q J R Meteorol Soc.

[30] Kalkstein LS, Valimont KM (1986). An evaluation of summer discomfort in the United States using a relative climatological index. Bull Am Meteorol Soc.

[31] Basu R, Hong C, Li D-K, Avalos LA (2017). The impact of maternal factors on the association between temperature and preterm delivery. Environ Res.

[32] Lajinian S, Hudson S, Applewhite L, Feldman J, Minkoff HL (1997). An association between the heat-humidity index and preterm labor and delivery: a preliminary analysis. Am J Public Health.

[33] Strand LB, Barnett AG, Tong S (2011). Methodological challenges when estimating the effects of season and seasonal exposures on birth outcomes. BMC Med Res Methodol.

[34] Thornton PE, Thornton MM, Mayer BW, Wilhelmi N, Wei Y, Devarakonda R, et al. Daymet: daily surface weather data on a 1-km grid for North America, Version 3. Oak Ridge National Lab.(ORNL), Oak Ridge, TN (United States); 2016. Available from: 10.3334/ORNLDAAC/1328

[35] Hardy B. ITS-90 formulations for vapor pressure, frost point temperature, dewpoint temperature, and enhancement factors in the range–100 to+ 100 C. The Proceedings of the Third International Symposium on Humidity & Moisture, April 1998. Teddington; 1998. p. 1–8.

[36] Sonntag D (1990). Important new values of the physical constants of 1986, vapour pressure formulations based on the ITS-90, and psychrometer formulae. Z Für Meteorol.

[37] Robinson PJ (2001). On the definition of a heat wave. J Appl Meteorol.

[38] Peng RD, Bobb JF, Tebaldi C, McDaniel L, Bell ML, Dominici F (2010). Toward a quantitative estimate of future heat wave mortality under global climate change. Environ Health Perspect.

[39] Tong S, Wang XY, Barnett AG (2010). Assessment of heat-related health impacts in Brisbane, Australia: comparison of different heatwave definitions. PLoS One.

[40] Xu Z, FitzGerald G, Guo Y, Jalaludin B, Tong S (2016). Impact of heatwave on mortality under different heatwave definitions: a systematic review and meta-analysis. Environ Int.

[41] Rothman KJ, Greenland S, Lash TL (2008). Chapter 8: case-control studies. Modern Epidemiology. Third edition. Lippincott Williams & Wilkins.

[42] Avalos LA, Chen H, Li D-K, Basu R. The impact of high apparent temperature on spontaneous preterm delivery: a case-crossover study. Environ Health. 2017;16(1):5. 10.1186/s12940-017-0209-5 PMC5286689 28143601

[43] Auger N, Naimi AI, Smargiassi A, Lo E, Kosatsky T (2014). Extreme heat and risk of early delivery among preterm and term pregnancies. Epidemiology..

[44] Khamis Y, Shaala S, Damarawy H, Romia A, Toppozada M (1983). Effect of heat on uterine contractions during normal labor. Int J Gynaecol Obstet.

[45] Vähä-Eskeli K, Erkkola R (1991). The effect of short-term heat stress on uterine contractility, fetal heart rate and fetal movements at late pregnancy. Eur J Obstet Gynecol Reprod Biol.

[46] Stan CM, Boulvain M, Pfister R, Hirsbrunner-Almagbaly P (2013). Hydration for treatment of preterm labour. Cochrane Database Syst Rev.

[47] Chersich MF, Pham MD, Areal A, Haghighi MM, Manyuchi A, Swift CP, et al. Associations between high temperatures in pregnancy and risk of preterm birth, low birth weight, and stillbirths: systematic review and meta-analysis. BMJ. 2020;371:m3811. 10.1136/bmj.m3811 PMC7610201 33148618

[48] Smith ML, Hardeman RR (2020). Association of summer heat waves and the probability of preterm birth in Minnesota: an exploration of the intersection of race and education. Int J Environ Res Public Health.

[49] U.S. Department of Commerce Bureau of the Census. American Housing Survey for the United States in 1985. Washington, D.C.: U.S. Department of Commerce Bureau of the Census; 1988.

[50] Asta F, Michelozzi P, Cesaroni G, De Sario M, Badaloni C, Davoli M (2019). The modifying role of socioeconomic position and greenness on the short-term effect of heat and air pollution on preterm births in Rome, 2001–2013. Int J Environ Res Public Health.

[51] Markevych I, Schoierer J, Hartig T, Chudnovsky A, Hystad P, Dzhambov AM, de Vries S, Triguero-Mas M, Brauer M, Nieuwenhuijsen MJ, Lupp G, Richardson EA, Astell-Burt T, Dimitrova D, Feng X, Sadeh M, Standl M, Heinrich J, Fuertes E (2017). Exploring pathways linking greenspace to health: theoretical and methodological guidance. Environ Res.

[52] Jesdale BM, Morello-Frosch R, Cushing L (2013). The racial/ethnic distribution of heat risk–related land cover in relation to residential segregation. Environ Health Perspect.

[53] Sun S, Weinberger KR, Spangler KR, Eliot MN, Braun JM, Wellenius GA (2019). Ambient temperature and preterm birth: a retrospective study of 32 million US singleton births. Environ Int.

[54] Coris EE, Ramirez AM, Van Durme DJ (2004). Heat illness in athletes. Sports Med.

[55] Perry AG, Korenberg MJ, Hall GG, Moore KM. Modeling and syndromic surveillance for estimating weather-induced heat-related illness. J Environ Public Health. 2011;2011:1:10. 10.1155/2011/750236 PMC3103938 21647355

[56] Armstrong B, Sera F, Vicedo-Cabrera AM, Abrutzky R, Åström DO, Bell ML, Chen BY, de Sousa Zanotti Stagliorio Coelho M, Correa PM, Dang TN, Diaz MH, Dung DV, Forsberg B, Goodman P, Guo YLL, Guo Y, Hashizume M, Honda Y, Indermitte E, Íñiguez C, Kan H, Kim H, Kyselý J, Lavigne E, Michelozzi P, Orru H, Ortega NV, Pascal M, Ragettli MS, Saldiva PHN, Schwartz J, Scortichini M, Seposo X, Tobias A, Tong S, Urban A, de la Cruz Valencia C, Zanobetti A, Zeka A, Gasparrini A (2019). The role of humidity in associations of high temperature with mortality: a multicountry, multicity study. Environ Health Perspect.

[57] Walfisch A, Kabakov E, Friger M, Sheiner E (2017). Trends, seasonality and effect of ambient temperature on preterm delivery. J Matern Fetal Neonatal Med.

