# Supplementary material for: Acute associations between heatwaves and preterm and early-term birth in 50 US metropolitan areas: a matched case-control study
Source: Environ Health. 2021 Apr 23;20:47. doi: 10.1186/s12940-021-00733-y (PMC8066488; doi:10.1186/s12940-021-00733-y)
Supplement: Supplementary file 1 — Additional file 1: Figure S1. Map of Los Angeles-Long Beach-Santa Ana MSA. The MSA was split into Los Angeles county (mint green) and Orange county (light orange). Figure S2. Map of New York-North New Jersey-Long Island MSA. The MSA was split into the Long Island area (blue), New York City area (pink), and other New York and New Jersey counties (green). Figure S3. Illustration of the selection of pregnancies whose at-risk windows overlap with the warm season for preterm (blue) and early-term birth (orange) where pregnancy 1-4 (four arrow lines) are extreme examples determining the range of the eligible LMP dates. The at-risk window was defined as 28 weeks and 0 days to 36 weeks and 6 days for preterm birth and 37 weeks and 0 days to 38 weeks and 6 days for early-term birth. Figure S4. Flow chart of identification of preterm and early-term cases and matching control. Table S1. Detailed model specification for the analyses of preterm and early-term birth for different types of heatwave indicators. Table S2. Odds ratio estimates of heatwave indicators based on definition framework 1 (97.5th percentile of temperature) on early-term birth matching on maternal race, maternal education, and location. Table S3. Odds ratio estimates of heatwave indicators based on definition framework 2 (85th percentile of positive excessive heat factor) on early-term birth matching on maternal race, maternal education, and location. Table S4. Odds ratio estimates of heatwave indicators based on definition framework 1 (97.5th percentile of temperature) on preterm birth matching on maternal race, maternal education, and location. Table S5. Odds ratio estimates of heatwave indicators based on definition framework 2 (85th percentile of positive excessive heat factor) on preterm birth matching on maternal race, maternal education, and location. Table S6. Odds ratio estimates of heatwave indicators based on definition framework 1 (97.5th percentile of temperature) on preterm birth matching on mat [file 12940_2021_733_MOESM1_ESM.docx]

Supplemental Material

**Title**

**Acute Associations between Heatwaves and Preterm and Early-term Birth in 50 US Metropolitan Areas: A Matched Case-Control Study**

**Authors**

Mengjiao Huang, Matthew J. Strickland, Megan Richards, Heather A. Holmes, Andrew J. Newman, Joshua V. Garn, Yan Liu, Joshua L. Warren, Howard H. Chang, Lyndsey A. Darrow

**Table of Contents**

[**Figure S1** Map of Los Angeles-Long Beach-Santa Ana MSA. The MSA was split into Los Angeles county (Mint green) and Orange county (Light orange). 3](#_Toc65140889)

[**Figure S2** Map of New York-North New Jersey-Long Island MSA. The MSA was split into the Long Island area (blue), New York City area (pink), and other New York and New Jersey counties (green). 3](#_Toc65140890)

[**Figure S3** Illustration of the selection of pregnancies whose at-risk windows overlap with the warm season for preterm (blue) and early-term birth (orange) where pregnancy 1-4 (four arrow lines) are extreme examples determining the range of the eligible LMP dates. The at-risk window was defined as 28 weeks and 0 days to 36 weeks and 6 days for preterm birth and 37 weeks and 0 days to 38 weeks and 6 days for early-term birth. 4](#_Toc65140891)

[**Figure S4** Flow chart of identification of preterm and early-term cases and matching control 5](#_Toc65140892)

[**Table S1** Detailed model specification for the analyses of preterm and early-term birth for different types of heatwave indicators 6](#_Toc65140893)

[**Table S2** Odds ratio estimates of heatwave indicators based on definition framework 1 (97.5^th^ percentile of temperature) on early-term birth matching on maternal race, maternal education, and location 7](#_Toc65140894)

[**Table S3** Odds ratio estimates of heatwave indicators based on definition framework 2 (85^th^ percentile of positive excessive heat factor) on early-term birth matching on maternal race, maternal education, and location 8](#_Toc65140895)

[**Table S4** Odds ratio estimates of heatwave indicators based on definition framework 1 (97.5^th^ percentile of temperature) on preterm birth matching on maternal race, maternal education, and location 9](#_Toc65140896)

[**Table S5** Odds ratio estimates of heatwave indicators based on definition framework 2 (85^th^ percentile of positive excessive heat factor) on preterm birth matching on maternal race, maternal education, and location 10](#_Toc65140897)

[**Table S6** Odds ratio estimates of heatwave indicators based on definition framework 1 (97.5^th^ percentile of temperature) on preterm birth matching on maternal race, maternal education, and month and year of the last menstrual period 11](#_Toc65140898)

[**Table S7** Odds ratio estimates of heatwave indicators based on definition framework 2 (85^th^ percentile of positive excessive heat factor) on preterm birth matching on maternal race, maternal education, and month and year of the last menstrual period 12](#_Toc65140899)

[**Table S8** Odds ratio estimates of heatwave indicators based on definition framework 1 (97.5^th^ percentile of temperature) on early-term birth matching on maternal race, maternal education, and month and year of the last menstrual period 13](#_Toc65140900)

[**Table S9** Odds ratio estimates of heatwave indicators based on definition framework 2 (85^th^ percentile of positive excessive heat factor) on early-term birth matching on maternal race, maternal education, and month and year of the last menstrual period 14](#_Toc65140901)

[**Table S10** Odds ratio estimates of heatwave indicators based on definition framework 1 (97.5^th^ percentile of temperature) on preterm birth matching on maternal race, maternal education, and location, excluding those with missing matching factors and covariates. 15](#_Toc65140902)

[**Table S11** Odds ratio estimates of heatwave indicators based on definition framework 1 (97.5^th^ percentile of temperature) on early-term birth matching on maternal race, maternal education, and location, excluding those with missing matching factors and covariates. 16](#_Toc65140903)

[**Figure S5**. Odds ratio estimates of heatwave indicators on early-term birth by maternal education (<12 years, 12 years, and ≥13 years) using mean temperature over 97.5^th^ percentile to quantify heatwave. P-values were calculated from the joint test for interaction. 17](#_Toc65140904)

[**Figure S6**. Odds ratio estimates of heatwave indicators on preterm birth by maternal education (<12 years, 12 years, and ≥13 years) using mean temperature over 97.5^th^ percentile to quantify heatwave. P-values were calculated from the joint test for interaction. 18](#_Toc65140905)

[**Figure S7**. Odds ratio estimates of heatwave indicators on early-term birth by child sex (male and female) using mean temperature over 97.5^th^ percentile to quantify heatwave. P-values were calculated from the joint test for interaction. 19](#_Toc65140906)

[**Figure S8.** Odds ratio estimates of heatwave indicators on preterm birth by child sex (male and female) using mean temperature over 97.5^th^ percentile to quantify heatwave. P-values were calculated from the joint test for interaction. 20](#_Toc65140907)

[**Figure S9**. Odds ratio estimates of heatwave indicators on early-term birth by region (Northeast, Midwest, South, and West) using mean temperature over 97.5^th^ percentile to quantify heatwave. P-values were calculated from the joint test for interaction. 21](#_Toc65140908)

[**Figure S10**. Odds ratio estimates of heatwave indicators on preterm birth by region (Northeast, Midwest, South, and West) using mean temperature over 97.5^th^ percentile to quantify heatwave. P-values were calculated from the joint test for interaction. 22](#_Toc65140909)


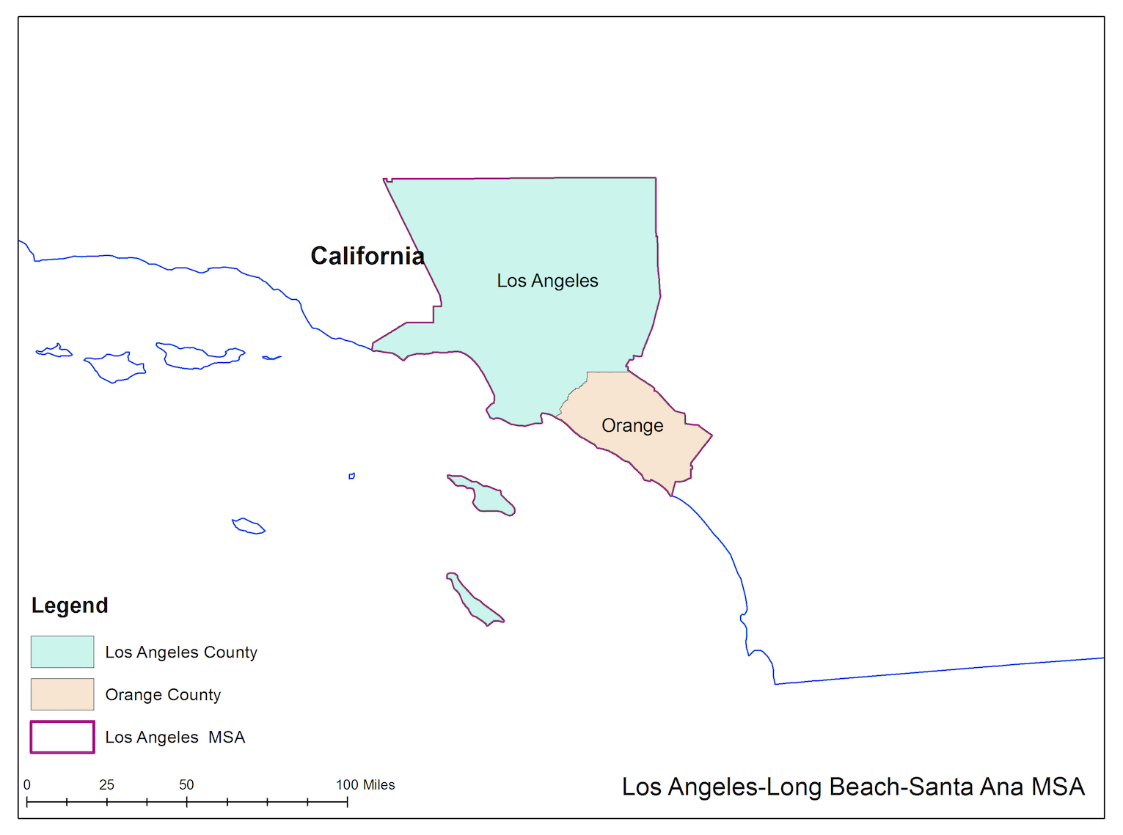


# Figure S1 Map of Los Angeles-Long Beach-Santa Ana MSA. The MSA was split into Los Angeles county (Mint green) and Orange county (Light orange).


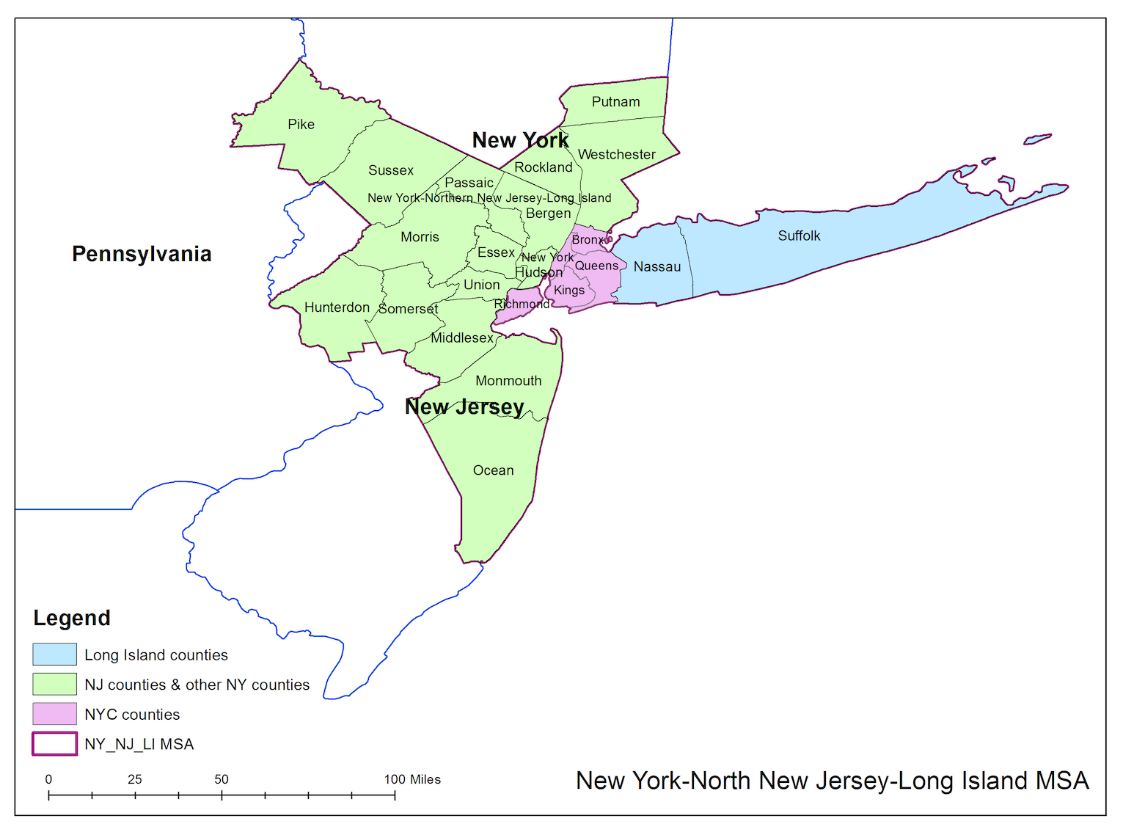


# **Figure S2** Map of New York-North New Jersey-Long Island MSA. The MSA was split into the Long Island area (blue), New York City area (pink), and other New York and New Jersey counties (green).

**At-risk window**

**Warm Season**

28w

36w6d

May 1^st^

Sep 30^th^

37w

**Pregnancy 2**

38w6d

**At-risk window**

**Pregnancy 1**

**Pregnancy** **3**

**Pregnancy 4**

Preterm

Early-term

Eligibility based on dates of last menstrual period (LMP): **Aug 16^th^ –Mar 18^th^ of the following year**

Eligibility based on dates of last menstrual period (LMP): **Aug 2^nd^–Jan 14^th^ of the following year**

# **Figure S3** Illustration of the selection of pregnancies whose at-risk windows overlap with the warm season for preterm (blue) and early-term birth (orange) where pregnancy 1-4 (four arrow lines) are extreme examples determining the range of the eligible LMP dates. The at-risk window was defined as 28 weeks and 0 days to 36 weeks and 6 days for preterm birth and 37 weeks and 0 days to 38 weeks and 6 days for early-term birth.

Gestation 37-38 w

24 cases could not be matched

2,011,152 observations

1,005,576 case-control pairs

11,441,225 singleton births

Twins or multiples (252,564)

A total of 13,174,372 births in 53 locations from 1982-1988

LMP dates were outside 01/10/1981-29/02/1988 (1,443,815)

11,693,789 births

Gestational period between 28w-36w+6d had no overlap with warm season: May 1^st^-Sep 30^th^ (4,300,657)

Gestation 28-36 w

≥37w

Missing date of birth and/or gestational age (36,768)

11,730,557 births

8 cases could not be matched

1,230,658 observations

615,329 case-control pairs

Preterm

Early- term

≥39w

Matching process: 1:1 matching on maternal education, race, and location (cumulative sampling)

Gestational period between 37w-38w+6d had no overlap with warm season: May 1^st^-Sep 30^th^ (5,887,676)

1,005,600 early-term cases

7,140,568 singleton births

5,553,549 singleton births

6,474,579 control pool

4,033,696 control pool

615,338 preterm cases

# **Figure S4** Flow chart of identification of preterm and early-term cases and matching control

# **Table S1** Detailed model specification for the analyses of preterm and early-term birth for different types of heatwave indicators

|  | **Model specification** |
| --- | --- |
| HW1 | $\mathrm{logit}\left[ P\left( outcome \right) \right]=\beta_{1}HW1_{cat1}+\beta_{2}HW1_{cat2}+\beta_{3}HW1_{cat3}+\beta_{4}marital status+\sum_{i=1}^{2} \beta_{5i}maternal age_{i}+\sum_{j=1}^{52} \beta_{6j}LMP\_m\_y_{j},$  where$HW1_{cat1}$, $HW1_{cat2}$, and $HW1_{cat3}$ are the dummy variables for HW1 category 1-3, $\beta_{1}$-$\beta_{3}$ are the regression coefficients for HW1 category 1-3. Model adjusted for maternal age (≤20, 21-34, ≥35 years), marital status (married vs. unmarried), and LMP month and year, and conditioned on the matched sets.  **Linear trend test:** $\mathrm{logit}\left[ P\left( outcome \right) \right]=\beta_{1}HW1+\beta_{4}marital status+\sum_{i=1}^{2} \beta_{5i}maternal age_{i}+\sum_{j=1}^{52} \beta_{6j}LMP\_m\_y_{j},$  where$HW1$ categories(0=1 , 1=1, 2=2, 3=3-7 days) were modeled as linear term to test whether β_1_ was significant (α=0.05) . |
| HW2_C2_ | $\mathrm{logit}\left[ P\left( outcome \right) \right]=\beta_{1}HW2_{C2}+\beta_{2}marital status+\sum_{i=1}^{2} \beta_{3i}maternal age_{i}+\sum_{j=1}^{52} \beta_{4j}LMP\_m\_y_{j},$  where HW2_C2_ was modeled as a binary variable (1=there was a ≥2 consecutive-day heatwave in the past week, 0=otherwise). Model adjusted for maternal age (≤20, 21-34, ≥35 years), marital status (married vs. unmarried), and LMP month and year, and conditioned on the matched sets. |
| HW2_C3_ | $\mathrm{logit}\left[ P\left( outcome \right) \right]=\beta_{1}HW2_{C3}+\beta_{2}marital status+\sum_{i=1}^{2} \beta_{3i}maternal age_{i}+\sum_{j=1}^{52} \beta_{4j}LMP\_m\_y_{j},$  where HW2_C3_ was modeled as a binary variable (1=there was a ≥3 consecutive-day heatwave in the past week, 0=otherwise). Model adjusted for maternal age (≤20, 21-34, ≥35 years), marital status (married vs. unmarried), and LMP month and year, and conditioned on the matched sets. |
| HW2_C4_ | $\mathrm{logit}\left[ P\left( outcome \right) \right]=\beta_{1}HW2_{C4}+\beta_{2}marital status+\sum_{i=1}^{2} \beta_{3i}maternal age_{i}+\sum_{j=1}^{52} \beta_{4j}LMP\_m\_y_{j},$  where HW2_C4_ was modeled as a binary variable (1=there was a ≥4 consecutive-day heatwave in the past week, 0=otherwise). Model adjusted for maternal age (≤20, 21-34, ≥35 years), marital status (married vs. unmarried), and LMP month and year, and conditioned on the matched sets. |
| HW3 | $\mathrm{logit}\left[ P\left( outcome \right) \right]=\beta_{1}HW3+\beta_{2}marital status+\sum_{i=1}^{2} \beta_{3i}maternal age_{i}+\sum_{j=1}^{52} \beta_{4j}LMP\_m\_y_{j},$  where HW3 was modeled as a continuous variable (°C for temperature or °C^2^ for EHF), β_1_ is the regression coefficient for 1 unit increase in HW3 value. Model adjusted for maternal age (≤20, 21-34, ≥35 years), marital status (married vs. unmarried), and LMP month and year, and conditioned on the matched sets. |
| ***Test for interaction*** | $\mathrm{logit}\left[ P\left( outcome \right) \right]=\sum_{i=1}^{x} \beta_{i}HW+\sum_{j=1}^{y} \gamma_{j}effect modifier+\sum_{k=1}^{(x*y)} \delta_{k}HW*effect modifier + \beta_{3}marital status+\sum_{m=1}^{2} \beta_{4i}maternal age_{i}+\sum_{n=1}^{52} \beta_{5j}LMP\_m\_y_{j},$  The joint test for the interaction terms was used to test differences between subgroups, (α=0.05) joint test null was that all δ=0. Effect modifiers examined included maternal race, maternal education, child sex, and region. Maternal age and child sex, which were not a matching factor in the primary analyses, were additionally matched when the effect modification was evaluated for these factors. |

# **Table S2** Odds ratio estimates of heatwave indicators based on definition framework 1 (97.5^th^ percentile of temperature) on early-term birth matching on maternal race, maternal education, and location

|  | **Tmin** | | **ATmin** | | **Tmax** | | **ATmax** | | **Tmean** | | **ATmean** | |
| --- | --- | --- | --- | --- | --- | --- | --- | --- | --- | --- | --- | --- |
|  | OR(95%CI) | P-value^*^ | OR(95%CI) | P-value^*^ | OR(95%CI) | P-value^*^ | OR(95%CI) | P-value^*^ | OR(95%CI) | P-value^*^ | OR(95%CI) | P-value^*^ |
| **HW1(total hot days)** |  |  |  |  |  |  |  |  |  |  |  |  |
| 0 | ref | <0.0001 | ref | <0.0001 | Ref | 0.0038 | ref | <0.0001 | ref | <0.0001 | ref | <0.0001 |
| 1 | 1.005(0.994, 1.016) |  | 1.006(0.996, 1.017) |  | 1.015(1.004, 1.026) |  | 1.016(1.004, 1.028) |  | 1.017(1.005, 1.029) |  | 1.007(0.995, 1.018) |  |
| 2 | 1.009(0.996, 1.022) |  | 1.005(0.991, 1.018) |  | 1.001(0.987, 1.015) |  | 1.003(0.989, 1.017) |  | 1.000(0.986, 1.013) |  | 1.009(0.995, 1.022) |  |
| 3+ | 1.033(1.020, 1.046) |  | 1.035(1.021, 1.048) |  | 1.018(1.006, 1.030) |  | 1.025(1.012, 1.039) |  | 1.027(1.014, 1.039) |  | 1.036(1.024, 1.049) |  |
|  |  |  |  |  |  |  |  |  |  |  |  |  |
| **HW2 (yes vs. no)** |  |  |  |  |  |  |  |  |  |  |  |  |
| C2 | 1.016(1.006, 1.026) |  | 1.018(1.008, 1.028) |  | 1.010(1.000, 1.020) |  | 1.015(1.005, 1.025) |  | 1.014(1.004, 1.024) |  | 1.023(1.013, 1.033) |  |
| C3 | 1.033(1.019, 1.047) |  | 1.035(1.021, 1.05) |  | 1.020(1.007, 1.032) |  | 1.025(1.012, 1.039) |  | 1.026(1.013, 1.039) |  | 1.032(1.019, 1.045) |  |
| C4 | 1.017(0.997, 1.037) |  | 1.022(1.002, 1.043) |  | 1.044(1.026, 1.063) |  | 1.035(1.017, 1.054) |  | 1.040(1.022, 1.059) |  | 1.027(1.008, 1.046) |  |
|  |  |  |  |  |  |  |  |  |  |  |  |  |
| **HW3 (continuous)** |  |  |  |  |  |  |  |  |  |  |  |  |
| per 1 °C | 1.029(1.006, 1.053) |  | 1.016(1.001, 1.031) |  | 1.022(1.004, 1.039) |  | 1.030(1.015, 1.045) |  | 1.045(1.022, 1.068) |  | 1.027(1.012, 1.042) |  |

*****P-value was calculated from the trend test across ordinal categories of HW1;

|  | **Tmin** | | **ATmin** | | **Tmax** | | **Atmax** | | **Tmean** | | **Atmean** | |
| --- | --- | --- | --- | --- | --- | --- | --- | --- | --- | --- | --- | --- |
|  | OR(95%CI) | P-value^*^ | OR(95%CI) | P-value^*^ | OR(95%CI) | P-value^*^ | OR(95%CI) | P-value^*^ | OR(95%CI) | P-value^*^ | OR(95%CI) | P-value^*^ |
| **HW1(total hot days)** |  |  |  |  |  |  |  |  |  |  |  |  |
| 0 | ref | <0.0001 | ref | <0.0001 | ref | 0.0019 | ref | <0.0001 | ref | <0.0001 | ref | <0.0001 |
| 1 | 1.015(0.997, 1.034) |  | 1.023(1.005, 1.042) |  | 1.027(1.007, 1.046) |  | 1.020(1.001, 1.040) |  | 1.036(1.016, 1.056) |  | 1.038(1.019, 1.058) |  |
| 2 | 1.028(1.005, 1.051) |  | 1.040(1.014, 1.066) |  | 1.004(0.984, 1.025) |  | 1.014(0.992, 1.036) |  | 1.012(0.991, 1.034) |  | 1.011(0.988, 1.034) |  |
| 3+ | 1.048(1.022, 1.074) |  | 1.040(1.015, 1.066) |  | 1.036(1.012, 1.061) |  | 1.053(1.029, 1.077) |  | 1.047(1.024, 1.072) |  | 1.058(1.034, 1.083) |  |
|  |  |  |  |  |  |  |  |  |  |  |  |  |
| **HW2 (yes vs. no)** |  |  |  |  |  |  |  |  |  |  |  |  |
| C2 | 1.036(1.018, 1.054) |  | 1.039(1.021, 1.058) |  | 1.013(0.997, 1.029) |  | 1.032(1.015, 1.048) |  | 1.027(1.010, 1.043) |  | 1.031(1.014, 1.048) |  |
| C3 | 1.044(1.018, 1.071) |  | 1.043(1.017, 1.069) |  | 1.036(1.011, 1.062) |  | 1.057(1.033, 1.082) |  | 1.049(1.025, 1.073) |  | 1.058(1.034, 1.083) |  |
| C4 | 1.085(1.034, 1.137) |  | 1.090(1.043, 1.140) |  | 1.030(0.990, 1.072) |  | 1.042(1.002, 1.083) |  | 1.062(1.019, 1.106) |  | 1.039(0.994, 1.087) |  |
|  |  |  |  |  |  |  |  |  |  |  |  |  |
| **HW3 (continuous)** |  |  |  |  |  |  |  |  |  |  |  |  |
| per 1 °C^2^ | 1.031(1.003, 1.060) |  | 1.018(1.004, 1.032) |  | 1.009(1.002, 1.015) |  | 1.017(1.008, 1.025) |  | 1.033(1.010, 1.056) |  | 1.023(1.009, 1.037) |  |

# **Table S3** Odds ratio estimates of heatwave indicators based on definition framework 2 (85^th^ percentile of positive excessive heat factor) on early-term birth matching on maternal race, maternal education, and location

*****P-value was calculated from the trend test across ordinal categories of HW1;

# **Table S4** Odds ratio estimates of heatwave indicators based on definition framework 1 (97.5^th^ percentile of temperature) on preterm birth matching on maternal race, maternal education, and location

|  | **Tmin** | | **ATmin** | | **Tmax** | | **ATmax** | | **Tmean** | | **ATmean** | |
| --- | --- | --- | --- | --- | --- | --- | --- | --- | --- | --- | --- | --- |
|  | OR(95%CI) | P-value^*^ | OR(95%CI) | P-value^*^ | OR(95%CI) | P-value^*^ | OR(95%CI) | P-value^*^ | OR(95%CI) | P-value^*^ | OR(95%CI) | P-value^*^ |
| **HW1(total hot days)** |  |  |  |  |  |  |  |  |  |  |  |  |
| 0 | ref | 0.37 | ref | 0.85 | ref | 0.02 | ref | 0.01 | ref | 0.03 | ref | 0.20 |
| 1 | 0.997(0.982, 1.012) |  | 0.993(0.978, 1.008) |  | 1.002(0.986, 1.018) |  | 1.013(0.996, 1.030) |  | 1.011(0.994, 1.028) |  | 1.005(0.988, 1.021) |  |
| 2 | 1.003(0.984, 1.023) |  | 1.006(0.987, 1.026) |  | 1.017(0.996, 1.037) |  | 1.011(0.991, 1.031) |  | 1.003(0.983, 1.023) |  | 1.015(0.995, 1.035) |  |
| 3+ | 1.010(0.992, 1.029) |  | 0.996(0.978, 1.015) |  | 1.018(1.000, 1.035) |  | 1.020(1.002, 1.037) |  | 1.020(1.003, 1.038) |  | 1.006(0.989, 1.025) |  |
|  |  |  |  |  |  |  |  |  |  |  |  |  |
| **HW2 (yes vs. no)** |  |  |  |  |  |  |  |  |  |  |  |  |
| C2 | 1.008(0.993, 1.022) |  | 1.002(0.988, 1.017) |  | 1.015(1.001, 1.029) |  | 1.013(0.999, 1.027) |  | 1.012(0.998, 1.026) |  | 1.009(0.995, 1.023) |  |
| C3 | 1.000(0.981, 1.020) |  | 0.999(0.979, 1.019) |  | 1.018(1.000, 1.037) |  | 1.014(0.995, 1.032) |  | 1.015(0.996, 1.033) |  | 1.009(0.990, 1.028) |  |
| C4 | 1.002(0.974, 1.032) |  | 1.004(0.975, 1.034) |  | 1.024(0.998, 1.050) |  | 1.018(0.993, 1.045) |  | 1.018(0.992, 1.044) |  | 1.018(0.992, 1.046) |  |
|  |  |  |  |  |  |  |  |  |  |  |  |  |
| **HW3 (continuous)** |  |  |  |  |  |  |  |  |  |  |  |  |
| per 1 °C | 0.980 (0.947, 1.015) |  | 0.987(0.965, 1.010) |  | 1.023(0.997, 1.048) |  | 1.018(0.996, 1.040) |  | 1.018(0.986, 1.050) |  | 1.007(0.986, 1.029) |  |

*****P-value was calculated from the trend test across ordinal categories of HW1;

# **Table S5** Odds ratio estimates of heatwave indicators based on definition framework 2 (85^th^ percentile of positive excessive heat factor) on preterm birth matching on maternal race, maternal education, and location

|  | **Tmin** | | **ATmin** | | **Tmax** | | **ATmax** | | **Tmean** | | **ATmean** | |
| --- | --- | --- | --- | --- | --- | --- | --- | --- | --- | --- | --- | --- |
|  | OR(95%CI) | P-value^*^ | OR(95%CI) | P-value^*^ | OR(95%CI) | P-value^*^ | OR(95%CI) | P-value^*^ | OR(95%CI) | P-value^*^ | OR(95%CI) | P-value^*^ |
| **HW1(total hot days)** |  |  |  |  |  |  |  |  |  |  |  |  |
| 0 | ref | 0.43 | ref | 0.30 | ref | 0.03 | ref | 0.06 | ref | 0.08 | ref | 0.45 |
| 1 | 1.015(0.989, 1.042) |  | 1.018(0.991, 1.045) |  | 1.000(0.973, 1.028) |  | 1.017(0.989, 1.046) |  | 1.002(0.975, 1.031) |  | 1.025(0.999, 1.055) |  |
| 2 | 0.998(0.965, 1.032) |  | 0.979(0.944, 1.016) |  | 1.024(0.995, 1.055) |  | 1.020(0.988, 1.053) |  | 1.005(0.974, 1.037) |  | 1.001(0.968, 1.035) |  |
| 3+ | 0.974(0.940, 1.010) |  | 0.978(0.943, 1.013) |  | 1.029(0.994, 1.065) |  | 1.020(0.987, 1.055) |  | 1.035(1.001, 1.069) |  | 1.005(0.972, 1.039) |  |
|  |  |  |  |  |  |  |  |  |  |  |  |  |
| **HW2 (yes vs. no)** |  |  |  |  |  |  |  |  |  |  |  |  |
| C2 | 0.985(0.961, 1.010) |  | 0.976(0.951, 1.002) |  | 1.027(1.004, 1.050) |  | 1.020(0.996, 1.044) |  | 1.021(0.998, 1.045) |  | 1.002(0.978, 1.027) |  |
| C3 | 0.979(0.943, 1.016) |  | 0.986(0.95, 1.023) |  | 1.025(0.990, 1.061) |  | 1.020(0.986, 1.055) |  | 1.036(1.002, 1.071) |  | 1.006(0.972, 1.041) |  |
| C4 | 0.964(0.899, 1.032) |  | 1.000(0.936, 1.067) |  | 1.039(0.982, 1.100) |  | 0.995(0.941, 1.052) |  | 1.043(0.984, 1.106) |  | 1.017(0.953, 1.085) |  |
|  |  |  |  |  |  |  |  |  |  |  |  |  |
| **HW3 (continuous)** |  |  |  |  |  |  |  |  |  |  |  |  |
| per 1 °C^2^ | 0.983(0.943, 1.025) |  | 0.982(0.962, 1.002) |  | 0.996(0.986, 1.006) |  | 0.996(0.984, 1.007) |  | 1.010(0.979, 1.042) |  | 0.999(0.981, 1.018) |  |

*****P-value was calculated from the trend test across ordinal categories of HW1;

# **Table S6** Odds ratio estimates of heatwave indicators based on definition framework 1 (97.5^th^ percentile of temperature) on preterm birth matching on maternal race, maternal education, and month and year of the last menstrual period

|  | **Tmin** | | **ATmin** | | **Tmax** | | **Atmax** | | **Tmean** | | **Atmean** | |
| --- | --- | --- | --- | --- | --- | --- | --- | --- | --- | --- | --- | --- |
|  | OR(95%CI) | P-value | OR(95%CI) | P-value | OR(95%CI) | P-value | OR(95%CI) | P-value | OR(95%CI) | P-value | OR(95%CI) | P-value |
| **HW1(total hot days)** |  |  |  |  |  |  |  |  |  |  |  |  |
| 0 | ref | 0.43 | ref | 0.58 | ref | 0.046 | ref | 0.02 | ref | 0.12 | ref | 0.11 |
| 1 | 1.004(0.989, 1.020) |  | 0.999(0.984, 1.015) |  | 1.004(0.987, 1.020) |  | 1.009(0.991, 1.026) |  | 1.012(0.994, 1.029) |  | 1.007(0.99, 1.024) |  |
| 2 | 1.011(0.991, 1.031) |  | 1.021(1.002, 1.042) |  | 1.016(0.995, 1.037) |  | 1.010(0.990, 1.031) |  | 1.001(0.981, 1.022) |  | 1.02(1, 1.041) |  |
| 3+ | 1.003(0.984, 1.022) |  | 0.996(0.977, 1.015) |  | 1.016(0.997, 1.034) |  | 1.021(1.002, 1.040) |  | 1.016(0.997, 1.035) |  | 1.009(0.99, 1.028) |  |
|  |  |  |  |  |  |  |  |  |  |  |  |  |
| **HW2 (yes vs. no)** |  |  |  |  |  |  |  |  |  |  |  |  |
| C2 | 1.005(0.989, 1.020) |  | 1.005(0.990, 1.021) |  | 1.013(0.999, 1.028) |  | 1.015(1.000, 1.030) |  | 1.009(0.994, 1.024) |  | 1.011(0.996, 1.026) |  |
| C3 | 0.998(0.978, 1.018) |  | 0.995(0.974, 1.016) |  | 1.013(0.995, 1.032) |  | 1.015(0.996, 1.034) |  | 1.006(0.987, 1.025) |  | 1.004(0.985, 1.024) |  |
| C4 | 0.998(0.969, 1.027) |  | 1.002(0.972, 1.032) |  | 1.001(0.975, 1.027) |  | 1.015(0.989, 1.042) |  | 1.005(0.979, 1.031) |  | 1.014(0.987, 1.042) |  |
|  |  |  |  |  |  |  |  |  |  |  |  |  |
| **HW3 (continuous)** |  |  |  |  |  |  |  |  |  |  |  |  |
| per 1 °C | 0.984(0.950, 1.019) |  | 0.994(0.972, 1.018) |  | 1.007(0.982, 1.033) |  | 1.009(0.987, 1.031) |  | 0.996(0.964, 1.029) |  | 1.002(0.981, 1.024) |  |

*****P-value was calculated from the trend test across ordinal categories of HW1;

# **Table S7** Odds ratio estimates of heatwave indicators based on definition framework 2 (85^th^ percentile of positive excessive heat factor) on preterm birth matching on maternal race, maternal education, and month and year of the last menstrual period

|  | **Tmin** | | **ATmin** | | **Tmax** | | **Atmax** | | **Tmean** | | **Atmean** | |
| --- | --- | --- | --- | --- | --- | --- | --- | --- | --- | --- | --- | --- |
|  | OR(95%CI) | P-value | OR(95%CI) | P-value | OR(95%CI) | P-value | OR(95%CI) | P-value | OR(95%CI) | P-value | OR(95%CI) | P-value |
| **HW1(total hot days)** |  |  |  |  |  |  |  |  |  |  |  |  |
| 0 | ref | 0.56 | ref | 0.35 | ref | 0.89 | ref | 0.32 | ref | 0.81 | ref | 0.91 |
| 1 | 1.015(0.989, 1.042) |  | 1.027(1.000, 1.054) |  | 0.994(0.967, 1.021) |  | 1.014(0.986, 1.043) |  | 1.001(0.974, 1.030) |  | 1.02(0.992, 1.049) |  |
| 2 | 0.995(0.962, 1.029) |  | 0.954(0.920, 0.991) |  | 1.014(0.984, 1.045) |  | 1.013(0.981, 1.046) |  | 0.999(0.968, 1.031) |  | 1.019(0.985, 1.054) |  |
| 3+ | 0.981(0.946, 1.017) |  | 0.991(0.956, 1.028) |  | 0.994(0.960, 1.029) |  | 1.008(0.975, 1.042) |  | 1.005(0.972, 1.039) |  | 0.983(0.950, 1.016) |  |
|  |  |  |  |  |  |  |  |  |  |  |  |  |
| **HW2 (yes vs. no)** |  |  |  |  |  |  |  |  |  |  |  |  |
| C2 | 0.986(0.961, 1.011) |  | 0.973(0.948, 0.999) |  | 1.007(0.984, 1.031) |  | 1.008(0.984, 1.033) |  | 1.003(0.979, 1.027) |  | 0.997(0.973, 1.022) |  |
| C3 | 0.988(0.951, 1.025) |  | 0.998(0.962, 1.036) |  | 0.997(0.963, 1.033) |  | 1.008(0.974, 1.043) |  | 1.008(0.975, 1.043) |  | 0.986(0.952, 1.020) |  |
| C4 | 1.011(0.942, 1.085) |  | 1.001(0.937, 1.070) |  | 0.970(0.917, 1.027) |  | 1.016(0.960, 1.076) |  | 1.023(0.964, 1.085) |  | 1.001(0.937, 1.069) |  |
|  |  |  |  |  |  |  |  |  |  |  |  |  |
| **HW3 (continuous)** |  |  |  |  |  |  |  |  |  |  |  |  |
| per 1 °C^2^ | 0.978(0.937, 1.021) |  | 0.977(0.957, 0.997) |  | 0.996(0.986, 1.006) |  | 0.996(0.984, 1.008) |  | 0.992(0.961, 1.023) |  | 0.994(0.975, 1.000) |  |

*****P-value was calculated from the trend test across ordinal categories of HW1;

# **Table S8** Odds ratio estimates of heatwave indicators based on definition framework 1 (97.5^th^ percentile of temperature) on early-term birth matching on maternal race, maternal education, and month and year of the last menstrual period

|  | **Tmin** | | **ATmin** | | **Tmax** | | **Atmax** | | **Tmean** | | **Atmean** | |
| --- | --- | --- | --- | --- | --- | --- | --- | --- | --- | --- | --- | --- |
|  | OR(95%CI) | P-value | OR(95%CI) | P-value | OR(95%CI) | P-value | OR(95%CI) | P-value | OR(95%CI) | P-value | OR(95%CI) | P-value |
| **HW1(total hot days)** |  |  |  |  |  |  |  |  |  |  |  |  |
| 0 | ref | <0.0001 | ref | <0.0001 | ref | <0.0001 | ref | <0.0001 | ref | <0.0001 | ref | <0.0001 |
| 1 | 1.005(0.994, 1.016) |  | 1.003(0.993, 1.014) |  | 1.009(0.998, 1.021) |  | 1.022(1.010, 1.034) |  | 1.015(1.003, 1.027) |  | 1.001(0.990, 1.012) |  |
| 2 | 1.021(1.007, 1.034) |  | 1.016(1.003, 1.029) |  | 1.005(0.991, 1.019) |  | 1.009(0.995, 1.024) |  | 1.01(0.996, 1.024) |  | 1.022(1.008, 1.036) |  |
| 3+ | 1.037(1.024, 1.051) |  | 1.042(1.028, 1.056) |  | 1.036(1.023, 1.048) |  | 1.041(1.028, 1.054) |  | 1.04(1.027, 1.053) |  | 1.046(1.033, 1.060) |  |
|  |  |  |  |  |  |  |  |  |  |  |  |  |
| **HW2 (yes vs. no)** |  |  |  |  |  |  |  |  |  |  |  |  |
| C2 | 1.024(1.014, 1.035) |  | 1.027(1.016, 1.037) |  | 1.023(1.012, 1.033) |  | 1.024(1.013, 1.034) |  | 1.023(1.013, 1.033) |  | 1.033(1.023, 1.043) |  |
| C3 | 1.035(1.021, 1.049) |  | 1.038(1.023, 1.053) |  | 1.032(1.019, 1.045) |  | 1.036(1.023, 1.050) |  | 1.036(1.023, 1.050) |  | 1.039(1.026, 1.053) |  |
| C4 | 1.024(1.004, 1.045) |  | 1.026(1.005, 1.047) |  | 1.055(1.036, 1.074) |  | 1.045(1.026, 1.064) |  | 1.045(1.027, 1.064) |  | 1.033(1.014, 1.053) |  |
|  |  |  |  |  |  |  |  |  |  |  |  |  |
| **HW3 (contious)** |  |  |  |  |  |  |  |  |  |  |  |  |
| per 1 °C | 1.034(1.010, 1.059) |  | 1.017(1.002, 1.033) |  | 1.034(1.016, 1.052) |  | 1.034(1.019, 1.050) |  | 1.053(1.030, 1.077) |  | 1.029(1.014, 1.044) |  |

*****P-value was calculated from the trend test across ordinal categories of HW1;

|  | **Tmin** | | **ATmin** | | **Tmax** | | **ATmax** | | **Tmean** | | **ATmean** | |
| --- | --- | --- | --- | --- | --- | --- | --- | --- | --- | --- | --- | --- |
|  | OR(95%CI) | P-value^*^ | OR(95%CI) | P-value^*^ | OR(95%CI) | P-value^*^ | OR(95%CI) | P-value^*^ | OR(95%CI) | P-value^*^ | OR(95%CI) | P-value^*^ |
| **HW1(total hot days)** |  |  |  |  |  |  |  |  |  |  |  |  |
| 0 | ref | <0.0001 | ref | <0.0001 | ref | 0.0003 | ref | <0.0001 | ref | <0.0001 | ref | <0.0001 |
| 1 | 1.028(1.010, 1.047) |  | 1.03(1.011, 1.048) |  | 1.026(1.007, 1.046) |  | 1.022(1.002, 1.042) |  | 1.043(1.023, 1.063) |  | 1.04(1.021, 1.060) |  |
| 2 | 1.021(0.998, 1.044) |  | 1.033(1.008, 1.060) |  | 1.023(1.002, 1.044) |  | 1.035(1.013, 1.058) |  | 1.028(1.006, 1.050) |  | 1.036(1.013, 1.061) |  |
| 3+ | 1.046(1.021, 1.073) |  | 1.040(1.015, 1.066) |  | 1.043(1.018, 1.068) |  | 1.049(1.025, 1.074) |  | 1.047(1.023, 1.072) |  | 1.054(1.030, 1.079) |  |
|  |  |  |  |  |  |  |  |  |  |  |  |  |
| **HW2 (yes vs. no)** |  |  |  |  |  |  |  |  |  |  |  |  |
| C2 | 1.030(1.013, 1.048) |  | 1.035(1.017, 1.054) |  | 1.027(1.011, 1.044) |  | 1.041(1.024, 1.058) |  | 1.036(1.019, 1.053) |  | 1.042(1.025, 1.060) |  |
| C3 | 1.042(1.016, 1.069) |  | 1.041(1.015, 1.067) |  | 1.040(1.014, 1.065) |  | 1.052(1.028, 1.077) |  | 1.044(1.020, 1.068) |  | 1.052(1.028, 1.077) |  |
| C4 | 1.110(1.058, 1.164) |  | 1.106(1.058, 1.157) |  | 1.058(1.016, 1.102) |  | 1.031(0.992, 1.072) |  | 1.059(1.016, 1.103) |  | 1.044(0.998, 1.091) |  |
|  |  |  |  |  |  |  |  |  |  |  |  |  |
| **HW3 (continuous)** |  |  |  |  |  |  |  |  |  |  |  |  |
| per 1 °C^2^ | 1.042(1.013, 1.072) |  | 1.022(1.008, 1.037) |  | 1.011(1.004, 1.018) |  | 1.012(1.004, 1.021) |  | 1.031(1.008, 1.054) |  | 1.019(1.005, 1.033) |  |

# **Table S9** Odds ratio estimates of heatwave indicators based on definition framework 2 (85^th^ percentile of positive excessive heat factor) on early-term birth matching on maternal race, maternal education, and month and year of the last menstrual period

*****P-value was calculated from the trend test across ordinal categories of HW1;

# **Table S10** Odds ratio estimates of heatwave indicators based on definition framework 1 (97.5^th^ percentile of temperature) on preterm birth matching on maternal race, maternal education, and location, excluding those with missing matching factors and covariates.

|  | **Tmin** | | **ATmin** | | **Tmax** | | **ATmax** | | **Tmean** | | **ATmean** | |
| --- | --- | --- | --- | --- | --- | --- | --- | --- | --- | --- | --- | --- |
|  | OR(95%CI) | P-value^*^ | OR(95%CI) | P-value^*^ | OR(95%CI) | P-value^*^ | OR(95%CI) | P-value^*^ | OR(95%CI) | P-value^*^ | OR(95%CI) | P-value^*^ |
| **HW1(total hot days)** |  |  |  |  |  |  |  |  |  |  |  |  |
| 0 | ref |  | ref |  | Ref |  | ref |  | ref |  | ref |  |
| 1 | 0.991(0.974, 1.009) | 0.39 | 0.985(0.968 ,1.003) | 0.74 | 1.009(0.990 ,1.028) | 0.02 | 1.020(1.000 ,1.04) | 0.01 | 1.015(0.995 ,1.035) | 0.03 | 1.003(0.984 ,1.022) | 0.23 |
| 2 | 0.992(0.971, 1.015) |  | 1.006(0.983 ,1.029) |  | 0.997(0.973 ,1.021) |  | 0.991(0.968 ,1.014) |  | 0.991(0.968 ,1.014) |  | 1.003(0.981 ,1.027) |  |
| 3+ | 1.001(0.980, 1.024) |  | 0.994(0.972 ,1.016) |  | 1.008(0.987 ,1.029) |  | 1.017(0.996 ,1.039) |  | 1.011(0.989 ,1.032) |  | 1.005(0.984 ,1.027) |  |
|  |  |  |  |  |  |  |  |  |  |  |  |  |
| **HW2 (yes vs. no)** |  |  |  |  |  |  |  |  |  |  |  |  |
| C2 | 1.001(0.984, 1.018) |  | 1.005(0.988 ,1.022) |  | 1.001(0.984 ,1.018) |  | 1.002(0.986 ,1.019) |  | 1.001(0.985 ,1.018) |  | 1.003(0.987 ,1.021) |  |
| C3 | 0.993(0.970, 1.017) |  | 1.000(0.976 ,1.024) |  | 1.012(0.990 ,1.034) |  | 1.010(0.988 ,1.032) |  | 1.006(0.984 ,1.029) |  | 1.008(0.985 ,1.031) |  |
| C4 | 0.996(0.962, 1.031) |  | 1.002(0.968 ,1.038) |  | 1.025(0.995 ,1.056) |  | 1.012(0.982 ,1.044) |  | 1.008(0.977 ,1.039) |  | 1.020(0.988 ,1.052) |  |
|  |  |  |  |  |  |  |  |  |  |  |  |  |
| **HW3 (continuous)** |  |  |  |  |  |  |  |  |  |  |  |  |
| per 1 °C | 0.981(0.941, 1.022) |  | 0.984(0.959 ,1.009) |  | 1.019(0.992 ,1.047) |  | 1.011(0.988 ,1.034) |  | 1.013(0.978 ,1.049) |  | 1.004(0.981 ,1.028) |  |

*****P-value was calculated from the trend test across ordinal categories of HW1;

# **Table S11** Odds ratio estimates of heatwave indicators based on definition framework 1 (97.5^th^ percentile of temperature) on early-term birth matching on maternal race, maternal education, and location, excluding those with missing matching factors and covariates.

|  | **Tmin** | | **ATmin** | | **Tmax** | | **ATmax** | | **Tmean** | | **ATmean** | |
| --- | --- | --- | --- | --- | --- | --- | --- | --- | --- | --- | --- | --- |
|  | OR(95%CI) | P-value^*^ | OR(95%CI) | P-value^*^ | OR(95%CI) | P-value^*^ | OR(95%CI) | P-value^*^ | OR(95%CI) | P-value^*^ | OR(95%CI) | P-value^*^ |
| **HW1(total hot days)** |  |  |  |  |  |  |  |  |  |  |  |  |
| 0 | ref |  | ref |  | Ref |  | ref |  | ref |  | ref |  |
| 1 | 1.011(0.999 ,1.023) | <.0001 | 1.009(0.997, 1.021) | <.0001 | 1.021(1.008, 1.035) | 0.004 | 1.015(1.002, 1.029) | 0.0001 | 1.021(1.007, 1.035) | <.0001 | 1.013(0.999, 1.026) | <.0001 |
| 2 | 1.014(0.999 ,1.03) |  | 1.007(0.991, 1.023) |  | 0.997(0.981, 1.014) |  | 1.001(0.985, 1.017) |  | 1.000(0.984, 1.016) |  | 1.005(0.989, 1.021) |  |
| 3+ | 1.048(1.031 ,1.064) |  | 1.048(1.032, 1.064) |  | 1.032(1.017, 1.048) |  | 1.036(1.021, 1.052) |  | 1.041(1.025, 1.057) |  | 1.055(1.039, 1.071) |  |
|  |  |  |  |  |  |  |  |  |  |  |  |  |
| **HW2 (yes vs. no)** |  |  |  |  |  |  |  |  |  |  |  |  |
| C2 | 1.024(1.011, 1.036) |  | 1.026(1.014, 1.039) |  | 1.016(1.004, 1.028) |  | 1.02(1.008, 1.032) |  | 1.020(1.007, 1.032) |  | 1.03(1.018, 1.042) |  |
| C3 | 1.046(1.029, 1.063) |  | 1.045(1.028, 1.063) |  | 1.033(1.018, 1.049) |  | 1.039(1.023, 1.055) |  | 1.039(1.023, 1.056) |  | 1.049(1.032, 1.065) |  |
| C4 | 1.036(1.011, 1.061) |  | 1.032(1.007, 1.057) |  | 1.057(1.035, 1.08) |  | 1.055(1.032, 1.078) |  | 1.049(1.027, 1.072) |  | 1.039(1.017, 1.062) |  |
|  |  |  |  |  |  |  |  |  |  |  |  |  |
| **HW3 (continuous)** |  |  |  |  |  |  |  |  |  |  |  |  |
| per 1 °C | 1.044(1.015, 1.074) |  | 1.018(1.000, 1.035) |  | 1.025(1.005, 1.045) |  | 1.036(1.02, 1.053) |  | 1.049(1.023, 1.075) |  | 1.028(1.012, 1.045) |  |

*****P-value was calculated from the trend test across ordinal categories of HW1;

**
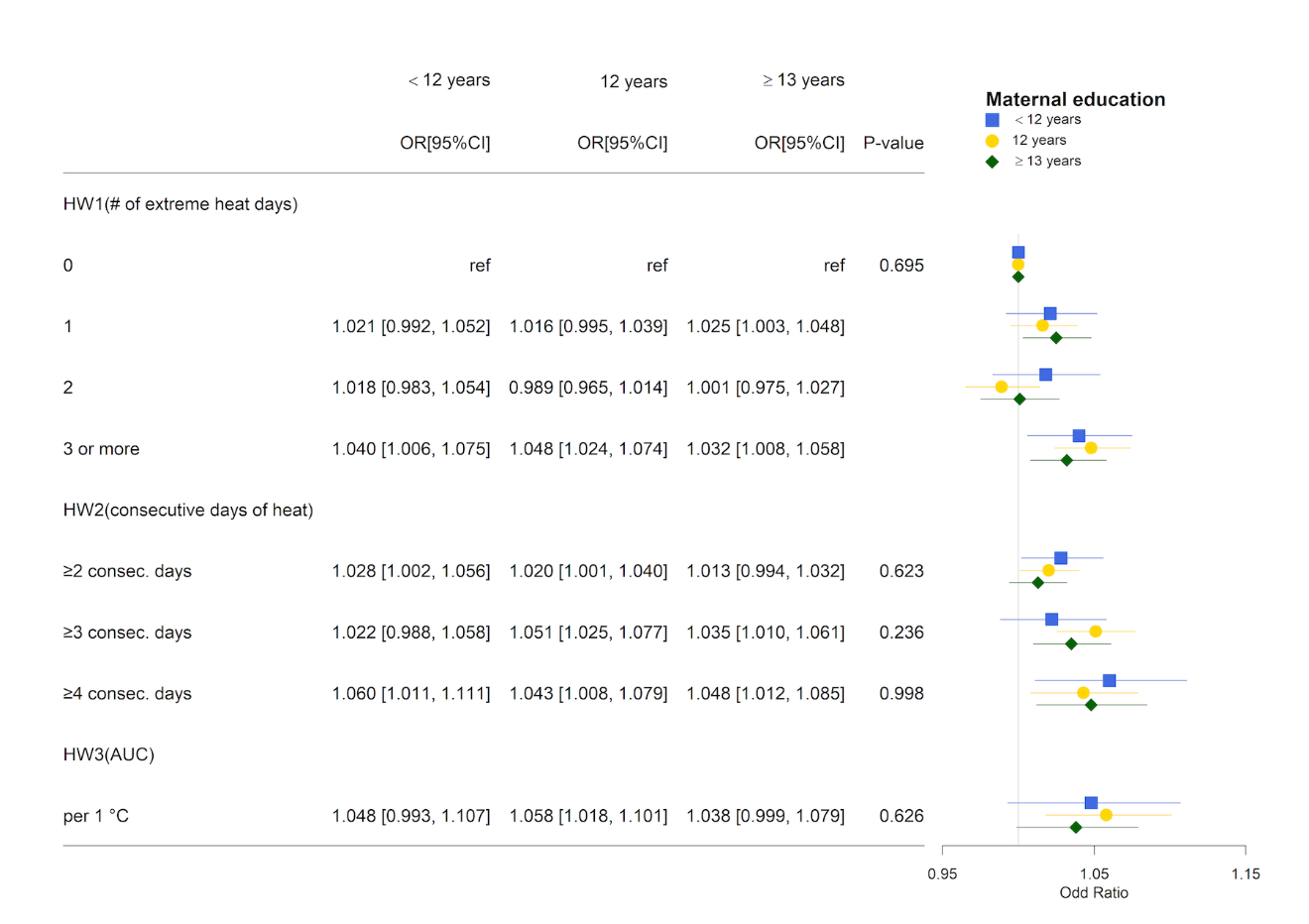
**

# **Figure S5**. Odds ratio estimates of heatwave indicators on early-term birth by maternal education (<12 years, 12 years, and ≥13 years) using mean temperature over 97.5^th^ percentile to quantify heatwave. P-values were calculated from the joint test for interaction.

**
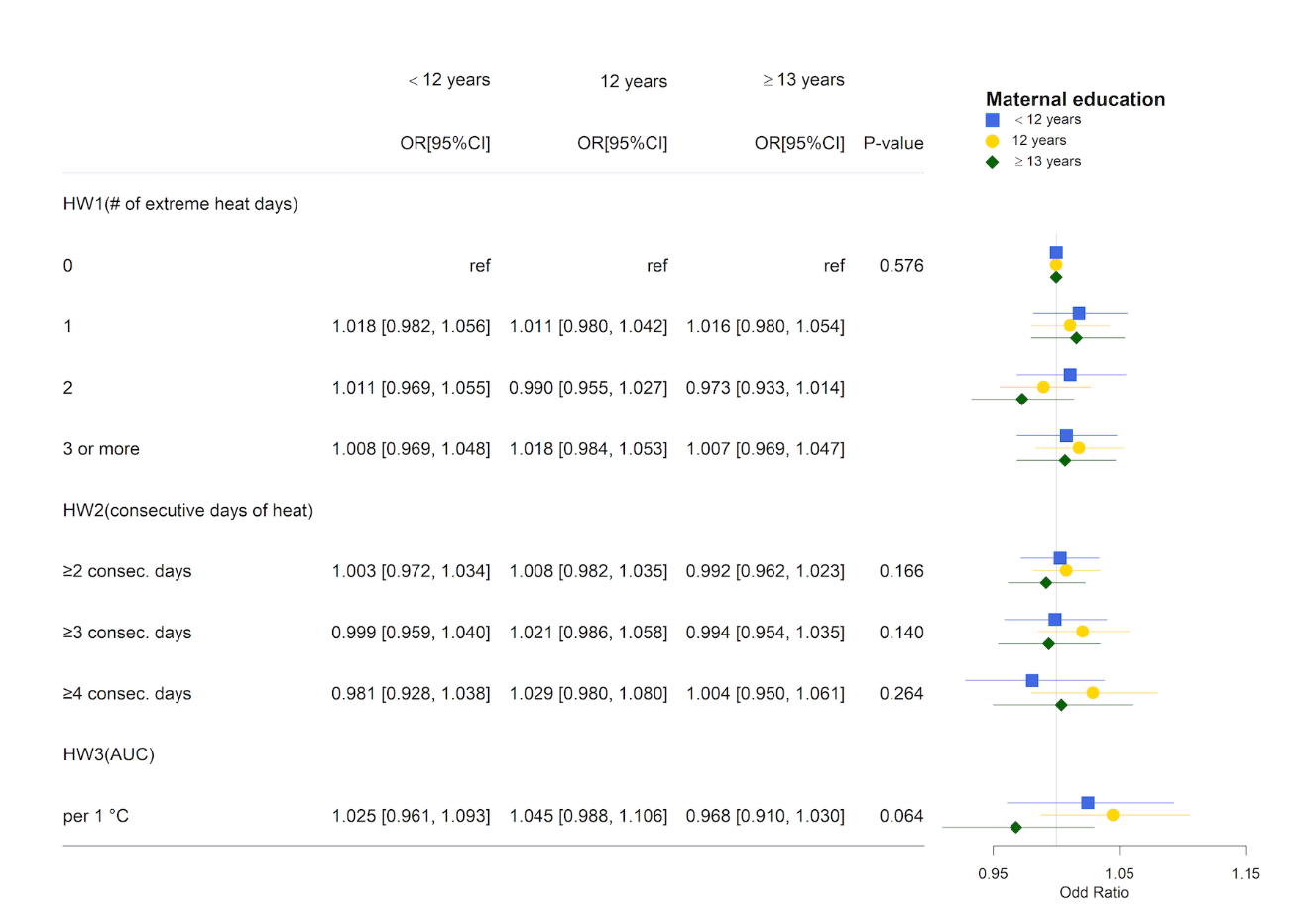
**

# **Figure S6**. Odds ratio estimates of heatwave indicators on preterm birth by maternal education (<12 years, 12 years, and ≥13 years) using mean temperature over 97.5^th^ percentile to quantify heatwave. P-values were calculated from the joint test for interaction.


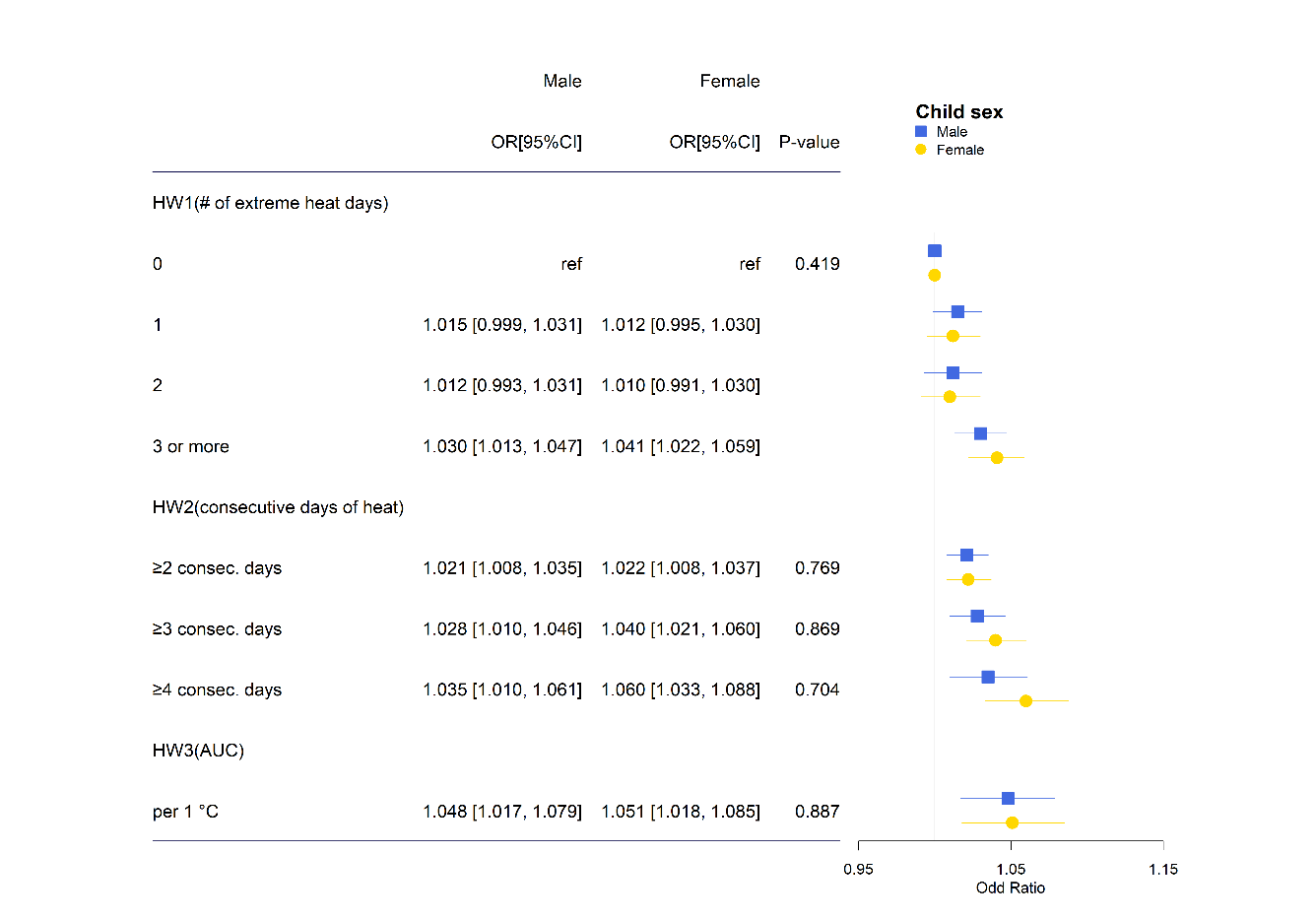


# **Figure S7**. Odds ratio estimates of heatwave indicators on early-term birth by child sex (male and female) using mean temperature over 97.5^th^ percentile to quantify heatwave. P-values were calculated from the joint test for interaction.


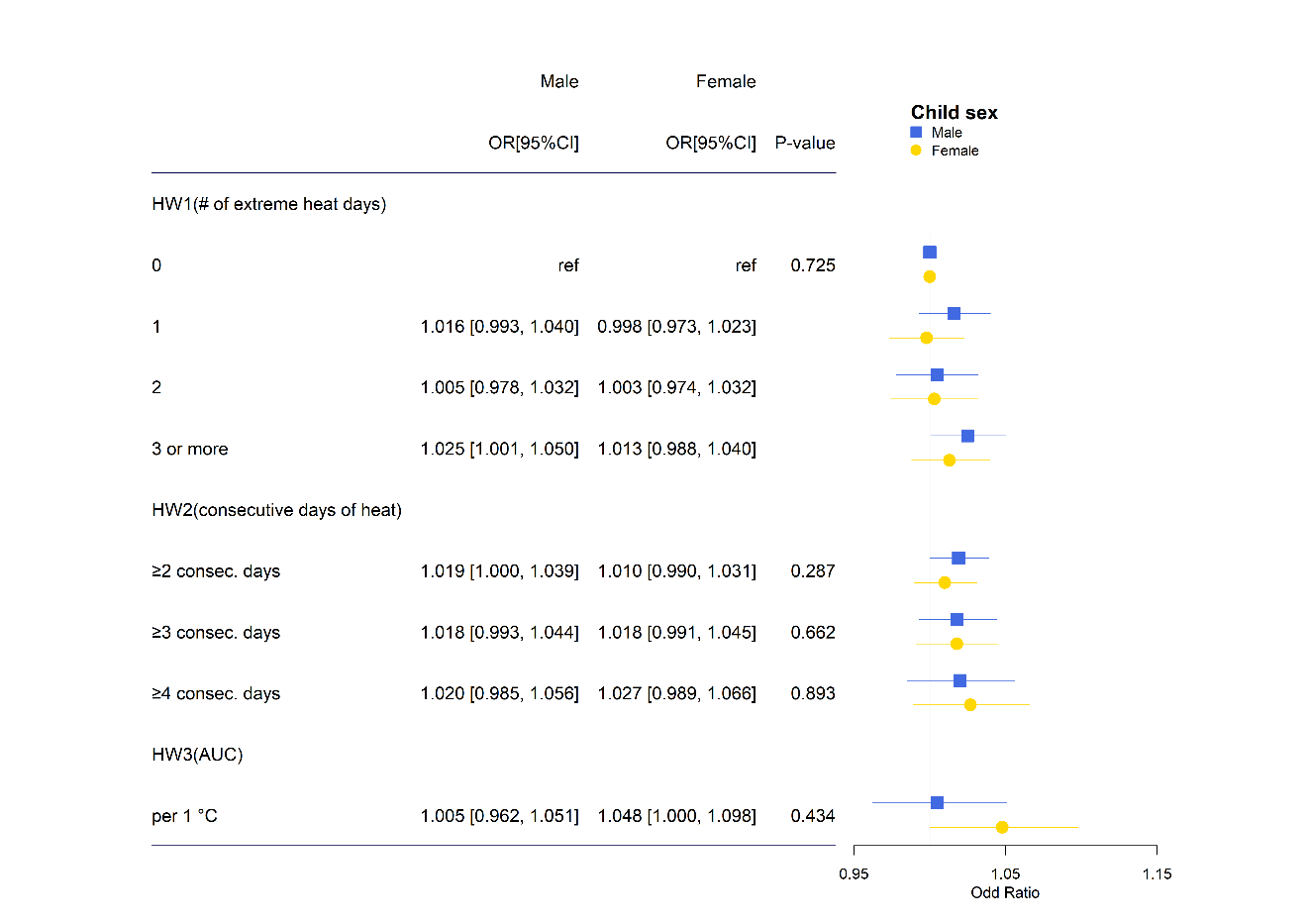


# **Figure S8.** Odds ratio estimates of heatwave indicators on preterm birth by child sex (male and female) using mean temperature over 97.5^th^ percentile to quantify heatwave. P-values were calculated from the joint test for interaction.


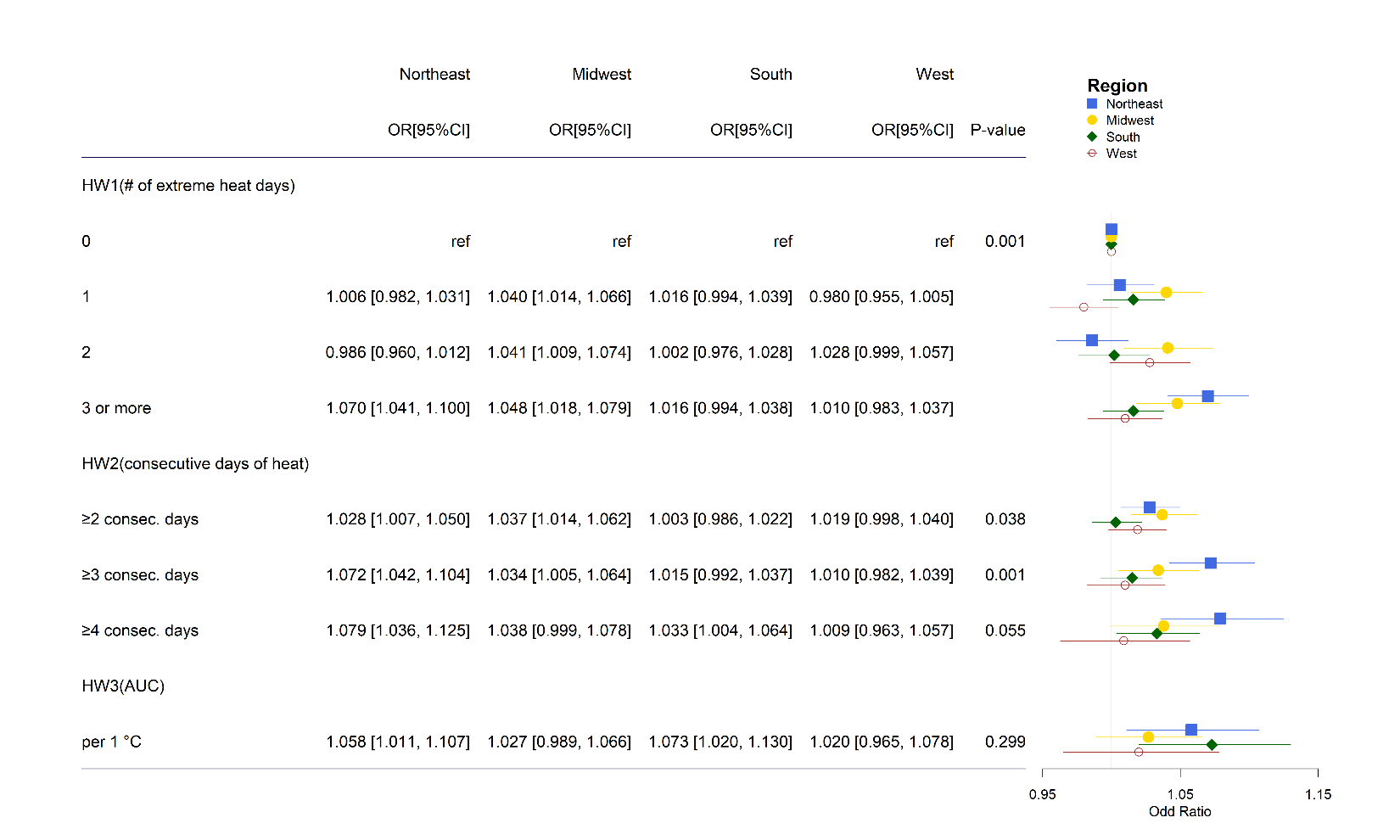


# **Figure S9**. Odds ratio estimates of heatwave indicators on early-term birth by region (Northeast, Midwest, South, and West) using mean temperature over 97.5^th^ percentile to quantify heatwave. P-values were calculated from the joint test for interaction.


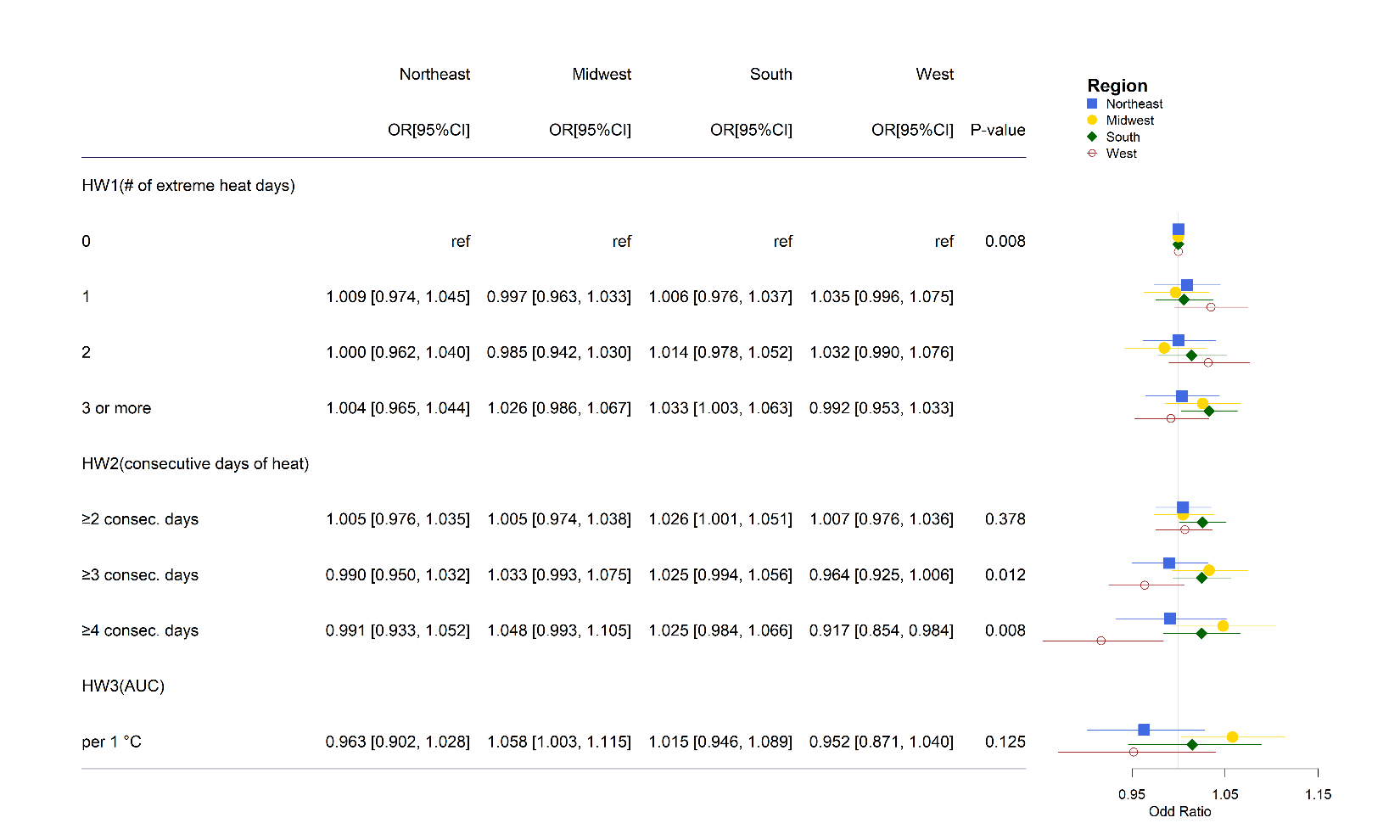


# **Figure S10**. Odds ratio estimates of heatwave indicators on preterm birth by region (Northeast, Midwest, South, and West) using mean temperature over 97.5^th^ percentile to quantify heatwave. P-values were calculated from the joint test for interaction.
